# The self-association equilibrium of DNAJA2 regulates its interaction with unfolded substrate proteins and with Hsc70

**DOI:** 10.1038/s41467-023-41150-8

**Published:** 2023-09-05

**Authors:** Lorea Velasco-Carneros, Jorge Cuéllar, Leire Dublang, César Santiago, Jean-Didier Maréchal, Jaime Martín-Benito, Moisés Maestro, José Ángel Fernández-Higuero, Natalia Orozco, Fernando Moro, José María Valpuesta, Arturo Muga

**Affiliations:** 1 Biofisika Institute (CSIC, UPV/EHU), University of the Basque Country, 48940 Leioa, Spain; 2 Department of Biochemistry and Molecular Biology, Faculty of Science and Technology, University of the Basque Country (UPV/EHU), 48940 Leioa, Spain; 3 Department of Macromolecular Structure, National Centre for Biotechnology (CNB-CSIC), 28049 Madrid, Spain; 4 Insilichem, Departament de Química, Universitat Autònoma de Barcelona, (UAB), 08193 Bellaterra (Barcelona), Spain

**Keywords:** Cryoelectron microscopy, Chaperones, Chaperones, Chaperones

## Abstract

J-domain proteins tune the specificity of Hsp70s, engaging them in precise functions. Despite their essential role, the structure and function of many J-domain proteins remain largely unknown. We explore human DNAJA2, finding that it reversibly forms highly-ordered, tubular structures that can be dissociated by Hsc70, the constitutively expressed Hsp70 isoform. Cryoelectron microscopy and mutational studies reveal that different domains are involved in self-association. Oligomer dissociation into dimers potentiates its interaction with unfolded client proteins. The J-domains are accessible to Hsc70 within the tubular structure. They allow binding of closely spaced Hsc70 molecules that could be transferred to the unfolded substrate for its cooperative remodelling, explaining the efficient recovery of DNAJA2-bound clients. The disordered C-terminal domain, comprising the last 52 residues, regulates its holding activity and productive interaction with Hsc70. These in vitro findings suggest that the association equilibrium of DNAJA2 could regulate its interaction with client proteins and Hsc70.

## Abstract (web summary)

J-domain proteins (JDPs) regulate Hsp70 function and specificity. Here, authors combine functional assays and cryoEM to describe the structure of a dynamic tubular assembly of DNAJA2, a class A JDP, and its stabilizing interdomain interactions.

## Introduction

Cell fitness depends on a correct balance between protein synthesis, folding and recycling. To ensure a healthy proteome, cells have devised a quality-control network that ensures folding, conformational maintenance and controlled degradation of proteins^1,2^. Molecular chaperones and cochaperones are essential since they arrange and interconnect these three main functions^3^.

The Hsp70 family of chaperones is an essential component of the cellular machinery engaged in the maintenance of protein homeostasis^4^. They are at the heart of the so-called Hsp70 system that includes two accessory proteins: a cochaperone that belongs to the J-domain protein (JDP) family, and a nucleotide exchange factor (NEF) that modulates the functional cycle of Hsp70^5,6^. This system regulates proteostasis through an ATP-dependent interaction with protein substrates^7^. The fact that there are more JDPs than Hsp70s in any organism or cellular compartment suggests that several JDPs can collaborate with a single Hsp70 to dictate its chaperone activity^8^. Therefore, the sequence and functional diversity of JDPs have been proposed to regulate many aspects of Hsp70 function including client specificity, localization, and ATPase/substrate remodelling activities^8–10^.

All Hsp70s contain a conserved, N-terminal ATPase domain (nucleotide binding domain, NBD) connected to the substrate binding domain (SBD) through a highly conserved linker^4^. In contrast, the variable domain composition of the members of the JDP family makes their classification difficult^8^. They are usually divided into three classes (A, B and C), each with a high degree of structural and functional variability^9^. Common to all of them is the presence of the J-domain (JD), an α-helical hairpin structure of 70–75 residues found at the N-terminus of A and B classes and anywhere in class C. In class A JDPs, the JD is followed by a flexible glycine/phenylalanine-rich region (G/FR), two homologous β sandwich domains (CTD I and II), with a Zn-finger-like region (ZFLR) inserted into the first one, a dimerization domain (DD), and a C-terminal domain (CD)^8^ (Supplementary Fig. 1a). All these domains but the ZFLR are also present in members of class B (Supplementary Fig. 1b), although some members of this class, such as DnaJB6, have distinct CTDs. In contrast, class C JDPs have a wide variety of different domains or functional motifs besides the JD. Beyond being obligate partners of Hsp70s, JDPs function by independently binding substrates and preventing their aggregation, an ability known as holding activity that requires regions other than the JD^11^.

High-resolution structures of different JDPs domains are available, although only one structure of a full-length class B JDP has been solved so far^12^, likely due to challenges resulting from the flexibility of these multidomain proteins^6^. Canonical class A and class B JDPs generally function as dimers^8,9^, albeit they can also transiently assemble into higher order homo/hetero oligomers to promote disaggregation^13^. Oligomers larger than dimers have been described for Class B JDPs such as DNAJB6 and DNAJB8^14–16^, and for the endoplasmic reticulum DNAJB11 that functions as a tetramer^17^. As for class A JDPs, a recently published study described the association of a representative of this protein family in *Streptococcus pneumonia* into oligomers^18^.

Despite the importance of JDPs in the functional guidance of Hsp70, a detailed structural characterization of class A members has not yet been performed. DNAJA2 has a potent holding activity, collaborates with Hsp70 in substrate remodelling and has been associated with other functions^8^. Structural knowledge of class A JDPs is necessary to understand how this chaperone family contributes to the functional diversification of the Hsp70 system. In this work, we use several biochemical and biophysical techniques to explore the functional and conformational properties of DNAJA2. In particular, we characterise its ability to self-assemble forming highly ordered, helical structures. These oligomers are stabilized by weak hydrophobic and electrostatic interactions. We obtain a three-dimensional cryo-electron microscopy (cryo-EM) structure of an ordered JDP oligomer. Further, we map the protein domains involved in oligomer formation and propose a functional role for the different oligomerisation states of the protein.

## Results

### DNAJA2 self-associates in solution

**Fig. 1:**
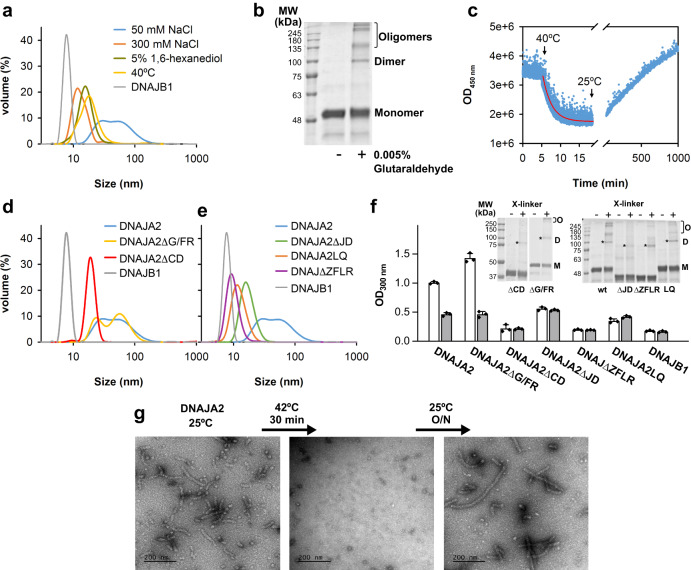
DNAJA2 dimers self-assemble into higher-order oligomers stabilized by multivalent interactions between different protein domains. **a** DLS experiments showing the volume against size distribution of 30 µM DNAJA2 (blue) or dimeric DNAJB1 (grey) in 20 mM Hepes pH 7.6, 2 mM DTT and 50 mM NaCl (blue). Addition of 300 mM NaCl (orange), 5% 1,5-hexanediol (green) or heat shock temperature (40 °C) (yellow) changed the size distribution of DNAJA2, suggesting oligomer dissociation. **b** Association state of DNAJA2 (2 µM) analysed by crosslinking with 0.005% glutaraldehyde (+) and SDS-PAGE. The same sample without crosslinker was also loaded in the gel (−). The different association states are indicated as monomer, dimer and oligomers, and were observed in three different protein batches. Left, molecular weight markers (MW). **c** Dissociation and reassociation of DNAJA2wt in response to stress temperature. Light scattered by a sample containing 15 µM DNAJA2 (blue) in refolding buffer (20 mM Hepes-KOH pH 7.6, 50 mM KCl, 5 mM MgCl_2_ and 2 mM DTT) heated to 40 °C and cooled back to 25 °C (temperature changes are marked with arrows). **d** Volume against size distribution of 30 µM DNAJA2wt (blue), DNAJA2ΔG/FR (yellow), DNAJA2ΔCD (red) and DNAJB1 (grey). **e** DLS experiments of 30 µM DNAJA2wt (blue), DNAJA2ΔJ (green), DNAJA2ΔZFLR (purple), DNAJA2LQ (yellow) and DNAJB1 (grey). **f** Light scattered at 300 nm by 15 µM DNAJA2wt or its mutants in the absence (white) or presence (grey) of 5% 1, 6-hexanediol. Data are means ± SD of three independent experiments. Inset, association states of the same samples followed by crosslinking with glutaraldehyde (+). The same samples without crosslinker were also loaded in the gel (−). M, monomer; D, dimer (marked with an asterisk) and O, oligomers. Left, molecular weight markers (MW). **g** EM analysis of the effect of temperature on the association equilibrium of DNAJA2wt. Negative staining images of DNAJA2wt (15 µM) treated as in c, showing that the ordered tubular oligomers formed at 25 °C dissociate at heat shock temperatures and reassociate upon cooling back the sample. These images are representative as they were observed in three different protein preparations.

To explore whether DNAJA2 oligomerises in solution, we characterised its association equilibrium by dynamic light scattering (DLS) and crosslinking experiments. DLS data revealed the presence of a heterogeneous population with a 20–100 nm size distribution that pointed to the dimeric DNAJA2 being able to assemble into larger oligomers (Fig. 1a). As a control of a dimeric J-domain proteins, DNAJB1, a member of the class B that lacks the CD but maintains the G/FR (Supplementary Fig. 1b), showed a single population with an average diameter of around 8 nm under the same experimental conditions (Fig. 1a), in accordance with the dimensions of a dimer estimated from a recent NMR structure^19^. In good agreement, crosslinking also showed that besides dimers, DNAJA2 could associate into higher order complexes (Fig. 1b). We next tested which type of interactions stabilised the oligomeric structure. To this aim, we treated the oligomer with the aliphatic alcohol 1,6-hexanediol, known to disrupt hydrophobic interactions involved in oligomer assembly^20^. We found that upon alcohol addition, the size distribution was displaced towards protein populations with a diameter of around 15 nm (Fig. 1a). This finding suggests that hydrophobic interactions stabilise the protein oligomer. The presence of an electrostatic component in the formation of these DNAJA2 assemblies was also evident by the decrease in particle size observed at 300 mM NaCl (Fig. 1a). Therefore, both hydrophobic and polar interactions are implicated in the formation of DNAJA2 oligomers. An additional factor regulating the association equilibrium was temperature, as under heat shock conditions (i.e. 40 °C), the equilibrium was shifted towards the dimer (Fig. 1a). Temperature-induced dissociation of the ordered tubular, oligomeric structures occurred in ~5 min (*k* = 0.43 min^−1^; *t*_1/2_ = 2.3 min) and the resulting dimers slowly reassociated into similar large assemblies at 25 °C (Fig. 1c).

### Several domains are involved in DNAJA2 oligomerisation

The domain composition of DNAJA2 shows a disordered C-terminal domain (CD) that comprises the final 52 residues (V_361_ to Q_412_) (Supplementary Fig. 1a). This region and the G/FR are predicted to be disordered by Metapredict^21^ (Supplementary Fig. 2a) and DISOPRED2 (Supplementary Fig. 2b), which gives a probability estimate of disorder for each residue in the sequence of a given protein^22^. MobiDB, a more restrictive algorithm that considers an intrinsically disordered region (IDR) when 20 consecutive residues are predicted to be disordered^23^, only predicts the last 48 residues to be IDR (Supplementary Fig. 2c). As IDRs are often involved in the interacting surface of protein-protein complexes^24^, we tested for their implication in cochaperone oligomerisation by generating deletion mutants of these domains (Supplementary Fig. 1b). DLS results showed that the mutant lacking the G/FR (DNAJA2ΔG/FR) was able to self-associate similarly to the wt protein. In contrast, truncation of the CD (DNAJA2ΔCD) induced oligomer dissociation, yielding particles with an average diameter compatible with a dimer of around 15 nm (Fig. 1d). In accordance with these data, only those protein species capable of self-associating (the wt protein and the DNAJA2ΔG/FR variant) were sensitive to 1,6-hexanediol, which induced a marked decrease of light scattering, whereas the stable dimer DNAJB1 and mutant DNAJA2ΔCD were not (Fig. 1f). Crosslinking experiments further underscored the impaired ability of this deletion mutant to self-assemble into large oligomers (Fig. 1f, inset).

We wanted to analyse in greater detail the structure of the cochaperone assembly, so we resorted to electron microscopy (EM). Initial observation of a DNAJA2 sample by negative staining revealed some small particles with a V-shape typical of the dimeric protein, in addition to the presence of temperature-sensitive, tubular structures ~24 nm wide and of variable length, up to 0.5 µm in some cases (Fig. 1g and Supplementary Fig. 3a). This value is significantly larger than the size distribution observed by DLS, suggesting that in solution the elongated structures could fragment or curl, thus resulting in a smaller apparent size. Despite this difference, both EM and DLS revealed the same domain-dependence of the self-association equilibrium. Negative staining of DNAJA2ΔG/FR and DNAJA2ΔCD confirmed in part the DLS experiments: whereas the DNAJA2ΔG/FR mutant showed the presence of tubular structures (Supplementary Fig. 3b) that could be associated with the oligomers detected by DLS, in the case of DNAJA2ΔCD only a very small percentage of assemblies were observed (Supplementary Fig. 3c). These results demonstrated that DNAJA2 generates large oligomers and that while G/FR is not involved in oligomer formation, the CD stabilises the oligomer.

### CryoEM structure of oligomeric DNAJA2

We next sought to analyse the oligomers in more detail using cryoEM. Aliquots of DNAJA2 were vitrified and visualised in a 200 keV FEI Talos Arctica, and the images confirmed the presence of ordered, cylindrical structures with a helical appearance (Supplementary Fig. 4a), but also the lack of rigidity already observed in the negatively stained specimens (Supplementary Fig. 3a). A total of 1482 movies were collected (Supplementary Table 1), and analysed using an approach that treated small areas of the cylindrical structures as single particles. Following this criterion, 49387 particles were selected and 2D classified (Supplementary Figs. 4b and 5).

After several rounds of 2D classification without imposing any kind of symmetry, the best classes were used for subsequent rounds of 3D classification. From the best reconstruction, a picture emerged of a hollow, cylindrical structure with ~200 Å width (Supplementary Fig. 4c). The configuration is that of a helix formed by the lateral interaction of five filaments produced by longitudinal contact between DNAJA2 dimers. The oligomeric structure could also be viewed as a stack of “disks,” each formed by five DNAJA2 dimers, which also revealed an internal symmetry between the two monomers of DNAJA2 dimers. Therefore, imposition of D_5_ symmetry generated a better, higher resolution structure of the oligomeric assembly (Supplementary Fig. 4d). From the structure, a clearer idea of the arrangement of the DNAJA2 dimer within the oligomer could be deduced: it is placed perpendicular to the long axis of the cylinder, with the DD pointing outwards, the CTDI and CTDII forming the body of the dimer, and the N-terminal region building the central cylinder (see circle in Supplementary Fig. 4d), which clearly has a role in maintenance of the oligomeric structure. Attempts to improve the quality of the 3D reconstruction were prevented by what we assumed was a great flexibility of the tubular structure, and the final resolution of the 3D reconstruction was 8.7 Å.

**Fig. 2:**
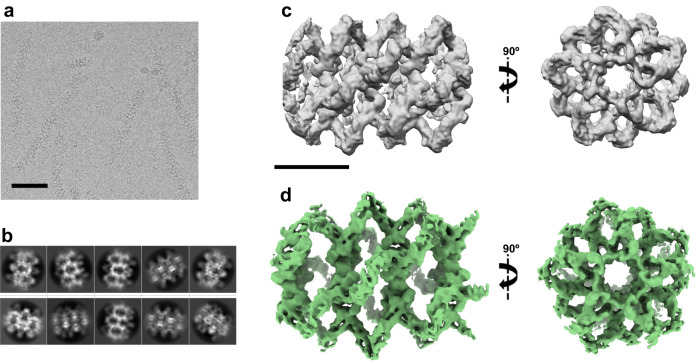
CryoEM structure of DNAJA2ΔG/FR. **a** A cryoEM field of DNAJA2ΔG/FR. Bar indicates 500 Å. **b** Maximum-likelihood 2D classification of the collected particles. **c** Side and top views of the 3D reconstruction of DNAJA2ΔG/FR without symmetry imposition. **d** Side and top views of the D5-symmetry imposed DNAJA2ΔG/FR map (6.9 Å resolution). Bar in (**c**) and (**d**) indicates 100 Å.

We directed our attention to the DNAJA2ΔG/FR mutant because a preliminary inspection of these oligomers suggested them to be less flexible (Supplementary Fig. 3b), possibly due to a lack of the disordered G/FR. This time, the cryogrids were first analysed in a 200 keV FEI Talos Arctica, and the best one used to record a total of 10714 movies on a 300 kV Titan Krios at the ESRF Grenoble facility (Supplementary Table 1). The DNAJA2ΔG/FR “tubes” (Fig. 2a) were again treated as single particles and segments of them were selected as such. With this approach, 560343 particles were selected and 2D classified (Fig. 2b), and the image processing and subsequent 3D reconstruction procedures are described in detail in the Methods section and in Supplementary Fig. 5. A preliminary 3D reconstruction without imposing symmetry yielded 9.2 Å resolution (Fig. 2c) and revealed the same general features observed for DNAJA2wt: an oligomeric structure in which the dimers associate in longitudinal filaments interacting laterally (~100 Å separation) to form a helical arrangement with ~32 Å pitch. The helical structure could also be visualised as disks of five DNAJA2ΔG/FR dimers, separated by ~76 Å.

Since the helical structure of the oligomers was obvious, we imposed helical symmetry on the DNAJA2ΔG/FR filaments using the protocol described in the Methods section. The volume generated resulted in an average Z raise of 32.25 Å and an average ϕ of 35.1° (Supplementary Fig. 4e). However, the high degree of heterogeneity due to the spring-like behaviour of the helices, with variations in the helical pitch and phi angle within the same helix (Supplementary Fig. 4f) resulted in a low-resolution 3D reconstruction. We finally imposed D_5_ symmetry in the longitudinal axis, which generated a better 3D reconstruction of 6.9 Å resolution (Fig. 2d). A clear picture emerged again of the arrangement of dimeric DNAJA2 within the oligomer: the DD is placed in the outer part of the structure, followed by the main body of the monomer (the CTDI, containing the ZFLR, and the CTDII), and at the core of the helical tube, with a very flexible arrangement, the JD.

**Fig. 3:**
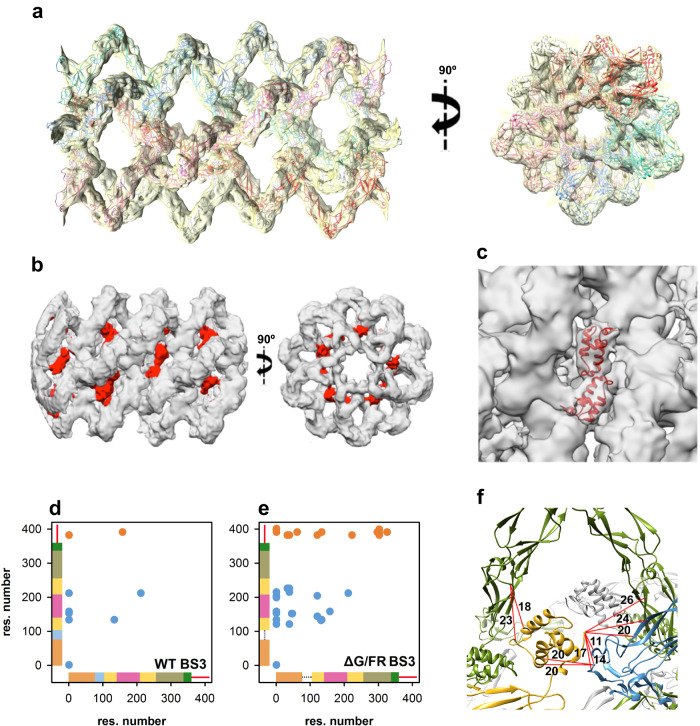
Atomic model of DNAJA2ΔG/FR in the cylindrical arrangement. **a** Side and top views of the atomic model of DNAJA2ΔG/FR fitted into the 3D reconstruction shown in Fig. 2d. Each filament that forms the oligomer is coloured differently. **b** Side and top views of the 3D reconstruction of DNAJA2ΔG/FR without symmetry imposition, highlighting the position of the JDs (in red). **c** Detail of two JDs of adjacent DNAJA2 monomers docked into the C1 DNAJA2ΔG/FR 3D reconstruction. **d**, **e** XL-MS contact maps of DNAJA2wt (**d**) and DNAJA2ΔG/FR (**e**) crosslinks identified using BS3 in conditions that favour the oligomer (20 mM NaCl and 20 °C). Blue dots correspond to inter-dimer crosslinks between domains included in the structural model derived from cryoEM (JD, CTDI, ZLFR and CTDII) and orange dots between the CD and other protein domains (see Supplementary Table 3). The axes are coloured to show the location of the JD (orange), G/FR (blue), CTDI (yellow), ZLFR (pink), CTDII (khaki), DD (green) and CD (red). **f** Inter-dimer crosslinks between Cα atoms of residues in the JD and amino acids in the CTDI and ZLFR mapped onto the C1 DNAJA2ΔG/FR 3D reconstruction. Crosslinks are shown as red lines and the distances measured in chimera are indicated in Å (see Supplementary Table 3).

To explore the role of each of these domains, an atomic model was generated using the sequence of DNAJA2ΔG/FR and the structures of the homologous DnaJA1 from yeast, Ydj1 (PDB IDs 1NLT and 1XAO, with a sequence identity of 49.6% and 40.4%, respectively) (Supplementary Table 2). The model of the DNAJA2 homodimer was refined by molecular dynamics (MD) and rigid body docking was performed to fit it into the 3D reconstruction. Docking was excellent with regard to the DD, CTDI and CTDII domains, whereas some movements were required to fit the ZFLR into the central cylinder of the tubular structure (Fig. 3a). Docking analysis revealed that, as expected from the disordered nature of its amino acid sequence, the CD located downstream from the DD is not visible. However, DLS and EM indicate that the CD is involved in oligomer stabilisation (Fig. 1d, f and Supplementary Fig. 3c). The length of this disordered stretch (52 residues) is enough to reach an adjacent DNAJA2 dimer, and thus it may play a yet unknown, albeit important role in the oligomerisation process. The CD occupies the outer part of the tubular oligomeric assembly and crosslinked CTDIs and JDs located in the inner region of this structure (Fig. 3d, e and Supplementary Table 3). This speaks of the CD’s ability to reach any part of the structure, including adjacent dimers within the ordered, oligomeric DNAJA2 structure.

A similar conclusion can be deduced for the JD. The following experimental findings engage the functionally essential JD in cochaperone oligomerisation, as its removal induces (i) the disappearance of the tubular structures (Supplementary Fig. 3d); (ii) a shift of the DLS profile towards smaller protein species with an estimated average diameter similar to that of DNAJA2wt in the presence of 1, 6-hexanediol (Fig. 1e); (iii) a lack of large molecular mass, inter-molecular crosslinks (Fig. 1f, inset); and (iv) the loss of sensitivity to 1,6-hexanediol (Fig. 1f). JDs have been proposed to have an important role in formation of transient complexes between different classes of JDPs, which provides a broad substrate specificity for the Hsc70 system in vitro and in cells^13,25,26^. Despite the clear role of JD in DNAJA2 oligomerisation, no mass in the 3D reconstruction could be attributed to this domain (Fig. 3a). The linker (13 residues) that connects the JD to the main body of the cochaperone in DNAJA2ΔG/FR is flexible enough to average out the JD during the 3D reconstruction process. A comparable behaviour was found for a deletion variant of class B DnaJ from *Thermus thermophilus*, which despite lacking the flexible region connecting the JD with the G/FR, showed different orientations of the JD in all four monomers within the asymmetric unit^12^. In the wt protein, the absence of electron density for the JD and G/FR indicated an even higher flexibility in this region^12^. Still, we managed to visualise the JDs using local masks in the region where they should be placed and without applying symmetry during the 3D reconstruction process, observing a clear mass of lower resolution than the rest of the 3D reconstructed volume (Fig. 3b, c). This lack of resolution can be explained by the flexibility of this domain, whose main role is to reach for and interact with Hsp70, stimulating its ATPase activity. In DNAJA2wt, the JD should be even more flexible due to the disordered G/FR (34 residues) that would allow the JD to protrude from the core of the helical structure. Crosslinks between the N-terminus and different protein regions also points to a conformational heterogeneity that allows the JD to adopt different orientations (Supplementary Table 3). The crosslinks of JDs with CTDIs and ZLFRs of two adjacent dimers are compatible with the proposed location of this flexible domain in the structural model (Fig. 3b, c, f and Supplementary Table 3), although alternative orientations of the JD within the ordered oligomers are possible. The increased number of crosslinks detected for the mutant could be rationalised if deletion of the disordered G/FR reduces the flexibility of the oligomeric structure.

### Localisation of the DNAJA2 oligomer-forming sites

**Fig. 4:**
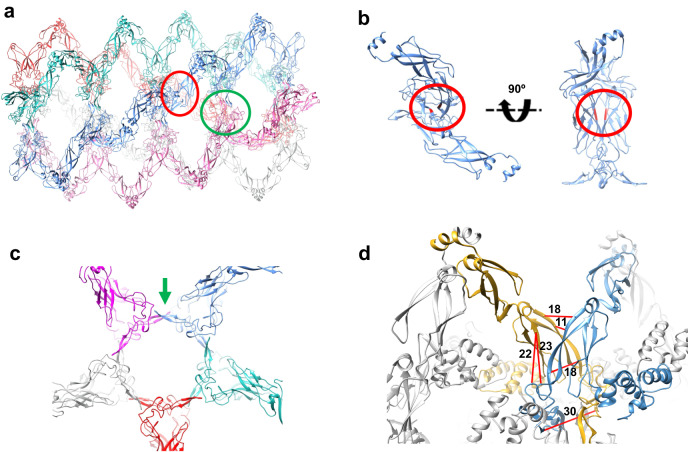
The CTD and ZFLR are engaged in the stabilisation of the oligomeric DNAJA2 assembly. **a** Side view of the atomic model of the DNAJA2ΔG/FR in which two DNAJA2 regions involved in the self-assembly process are highlighted. The first one (red circle) is localised at the core of CTDI and involves residues L135Q136 of monomers in adjacent dimers; the second one (green circle) involves the β-strand-loop-β−strand motif at the tip of ZFLR. **b** Zoom of the longitudinal interaction of DNAJA2 dimers at the CTDI highlighting residues L135Q136 (in red). **c** The view in (**b**) is rotated 90° and sliced so that only a “disk” of DNAJA2 is visualised. The green arrow points to the interaction between the β-strand-loop-β-strand motifs at the tip of two opposite ZFLRs. **d** Inter-dimer crosslinks between Cα atoms of residues in the CTDI and amino acids in the ZLFR mapped onto the DNAJA2ΔG/FR structural model. Crosslinks are shown as red lines and the distances measured in chimera are indicated in Å (see Supplementary Table 3).

The atomic model allowed analysis of the protein regions that stabilised the oligomer, and highlighted two important for the self-assembly process (Fig. 4a). The first seems to be related to the longitudinal interaction of DNAJA2 dimers that could lead to filament formation. It is located in the core region of the CTDI implicated in substrate interaction^19,27^, and involves residues L_135_Q_136_ of monomers in adjacent dimers within the same filament (red circles in Fig. 4b). The second one is located in the ZFLR and is responsible for the lateral interaction between filaments and therefore for their stabilisation in a helical arrangement, forming the disks that build the cylindrical structure (pointed by the green arrow in Fig. 4c). Although other regions of this domain may be involved in disk formation, it is clear that the β-strand-loop-β-strand motif at the “tip” of the domain is engaged in stabilisation of the circular structure through a planar β-sheet structure made up of a lateral, antiparallel interaction between two β-strand-loop-β-strand motifs (Fig. 4c).

To confirm our hypothesis that the two regions described above are involved in oligomer formation, two DNAJA2 mutants were generated: one with L_135_Q_136_ mutated to Glu (DNAJA2LQ) and another lacking the ZFLR (DNAJA2ΔZFLR) (Supplementary Fig. 1b). Neither of these mutants could form oligomeric particles, as seen by DLS (Fig. 1e). The average diameter of the variants was around 10 nm (DNAJA2LQ) and 9 nm (DNAJA2ΔZFLR). Truncation of the ZFLR rendered a dimeric particle similar in size to the homologous class B JDP DNAJB1 (8 nm; Fig. 1e), which naturally lacks the ZFLR. Crosslinking experiments supported this interpretation, as neither mutant significantly generated adducts corresponding to oligomers any larger than dimers. Transient interactions between dimers could generate some crosslinked species, significantly less abundant than in the wt protein (Fig. 1f, inset). These mutants were not sensitive to 1,6-hexanediol, unlike the DNAJA2wt and DNAJA2ΔG/FR (Fig. 1f). Finally, a visual inspection of these samples by negative-stain EM confirmed the absence of oligomers (Supplementary Fig. 3e, f). Interestingly, in the case of the DNAJA2ΔZFLR mutant, a certain percentage of filaments were observed that could be attributed to the preservation of the L_135_Q_136_ interaction between the CTDI of two adjacent dimers, (broken ellipses in Supplementary Fig. 3f). The intermolecular crosslink K134/K134, which according to the structural model is likely inter-dimeric (Supplementary Table 3), would further support their close proximity. Mapping the crosslinks into the structural model of the oligomer also suggested other contacts between CTDIs/ZLFRs of adjacent dimers that form the central core of the tubular structure (Fig. 4d and Supplementary Table 3). All these data confirm the importance of L_135_Q_136_ and the ZFLR in the formation and stabilisation of the oligomeric, helicoidal structure. Overall, it appears that interactions between JDs, CTDI(ZFLR)s and CDs stabilise the ordered, tubular structure.

### Functionality of the DNAJA2 variants: holding activity and collaboration with Hsc70

**Fig. 5:**
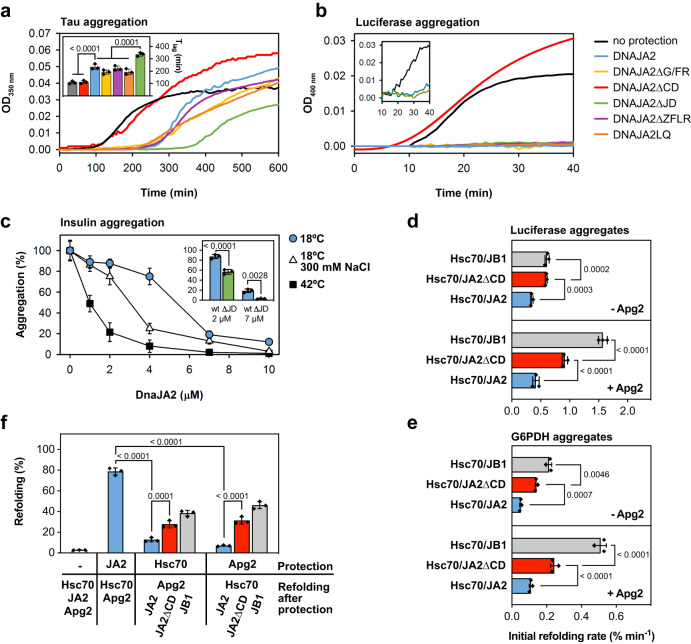
Functional characterization of DNAJA2 and its mutants. **a** Holding activity of the different DNAJA2 species. Light scattered at 350 nm by samples containing equimolar amounts (20 µM) of tau K18 P301L and heparin in the absence (black) or presence of 6 µM JDPs. Inset shows the estimated lag times for tau aggregation. **b** Aggregation of luciferase (0.125 µM) denatured at 42 °C in the absence (black) or presence of 1 µM JDP determined by light scattering at 400 nm. Inset shows aggregation of luciferase (0.125 µM) in the absence (black) or presence of 2 mM ATP and 2 µM Hsc70 (green) or 0.4 µM Apg2 (grey). The colour code used for the DNAJA2 variants in a and b is shown in panel (**b**). **c** Endpoints of insulin aggregation assays performed at 18 °C/0 mM NaCl (blue circles), 18 °C/300 mM NaCL (white triangles) or 42 °C/0 mM NaCl (black squares). Inset, comparison of the holding activity of DNAJA2wt (blue) and DNAJA2ΔJD (green) at 18 °C/0 mM NaCl. Aggregation of insulin (45 µM in 40 mM Hepes/KOH pH 7.5) was induced by the addition of 15 mM freshly prepared DTT and measured during 120 min. The corresponding kinetics are shown in Supplementary Fig. 6. Refolding rate (% refolded/min) of aggregates of (**d**) chemically denatured luciferase (6 M urea) or (**e**) thermally denatured G6PDH (42 °C) by 2 µM Hsc70, 0.4 µM Apg2 and 0.5 µM DNAJB1 (grey), DNAJA2ΔCD (red) or DNAJA2wt (blue). **f** Recovery of luciferase denatured (42 °C, 40 min) in the presence of 1 µM DNAJA2, 2 µM Hsc70 or 0.4 µM Apg2. After denaturation, 2 mM ATP and the missing chaperone components were added to end in all cases with 1 µM DNAJB1 (grey), DNAJA2ΔCD (red) or DNAJA2wt (blue), 0.4 µM Apg2 and 2 µM Hsc70, and recovery was measured after 120 min at 30 °C. Data are mean ± SD of three independent experiments. Significance was evaluated using a two-tailed, one-way analysis of variance (ANOVA) and Tukey’s multiple comparison, and the *p* values are shown in (**a**), (**c**–**f**).

We wanted to analyse the functional properties of the different DNAJA2 variants and of the wt protein under conditions that favour the dimeric or oligomeric species. To address this question, we first analysed the holding activity of the DNAJA2 variants using as substrate proteins tau K18P301L and luciferase. In the first case, DNAJA2ΔG/FR, DNAJA2ΔZFLR and DNAJA2LQ had a protecting role similar to DNAJA2wt (Fig. 5a). In contrast, DNAJA2ΔJD was more efficient in delaying the lag phase of tau aggregation, and removal of the disordered CD (DNAJA2ΔCD) abolished the ability to protect this substrate against aggregation (Fig. 5a). The holding activity of DNAJA2wt prevented aggregation of thermally denatured luciferase, as also seen for the protein variants except DNAJA2ΔCD (Fig. 5b), in good agreement with data obtained with tau. It is intriguing that deletion of the ZFLR or mutations in the CTDI, which are known to interact with substrates^19,28^, did not significantly affect the protein holding activity. This could be due to the presence of other protein regions (CTDII, remaining residues in the CTDI and likely the CD) engaged in client binding, which could compensate these changes^19^. Taken together, these results point to the CD as a critical region for the holding activity of the cochaperone.

To explore whether the association state of DNAJA2wt regulates its interaction with unfolded protein substrates, we used insulin as client protein because its aggregation can be induced by DTT^29^. Experiments were carried out under experimental conditions that favour the oligomer (low temperature and salt concentration; 18 °C/0 mM NaCl) or the dimer (high temperature or salt concentration; 42 °C/0 mM NaCl or 18 °C/300 mM NaCl) (Fig. 1a). Data indicated that the dimer induced at 300 mM NaCl or 42 °C displayed a stronger holding activity than the protein in conditions favouring the oligomer, especially evidenced below 7 μM cochaperone (Fig. 5c and Supplementary Fig. 6). They also suggested that at higher concentrations the oligomer can suppress substrate aggregation, albeit less efficiently than the dimer, most likely due to intermolecular interactions between domains that besides being involved in client binding are also engaged in protein oligomerisation (CTDIs and likely CDs). Insulin aggregation experiments reinforced the stronger holding activity of the DNAJA2ΔJD variant (inset to Fig. 5c and Supplementary Fig. 6d), in accordance with data obtained with tau.

We next tested the ability of the mutants to collaborate with Hsc70 using two types of experiments. First, following the stimulation of the Hsc70’s ATPase activity in the absence and presence of Apg2, an Hsp110 isoform with nucleotide exchange activity that is part of the Hsc70 system. Second, analysing their productive interaction with the chaperone in substrate remodelling. Activation experiments showed that in the absence of Apg2, all mutants but DNAJA2ΔCD stimulated Hsc70 slightly less efficiently than DNAJA2wt (Supplementary Fig. 7a). Differences between the DNAJA2 variants were clearly observed upon NEF addition. Under these conditions, three variants (DNAJA2ΔG/FR, DNAJA2ΔZFLR and DNAJA2LQ) failed to be as effective, albeit to a different extent, as the full-length protein, in contrast to DNAJA2ΔCD that was slightly better than DNAJA2wt in activating Hsc70 (Supplementary Fig. 7b). Titration of Apg2 with DNAJA2 (0–5 μM) did not modify the NEF´s ATPase activity (0.7 μmol ATP/min × μΜ Apg2) and therefore the activation observed for the ternary chaperone mixture (Hsc70/DNAJA2/Apg2) was only due to Hsc70. The following findings also suggested that recognition of the IDRs of DNAJA2 (G/FR and CD) by Hsc70 as pseudosubstrates that could activate the chaperone is not likely. First, a recent NMR study has shown that residues of the G/FR do not participate in Hsc70 binding^30^. Second, the ATPase activity of Hsc70 was not affected by a high concentration (80 μM) of isolated CD (Supplementary Fig. 7c). Therefore, the G/FR and ZFLR are required for full activation of Hsc70, whereas the CD is not.

Productive interaction of the DNAJA2 variants with Hsc70 in substrate remodelling was analysed using aggregates of luciferase and G6PDH. The DNAJA2ΔG/FR and DNAJA2ΔZFLR variants were virtually inactive regardless of the presence of Apg2 (Supplementary Fig. 7c, d). In contrast, the disaggregating/refolding activity of DNAJA2LQ was strongly dependent on Apg2, as it was reduced by ~60% in the presence of the NEF and negligible in its absence (Supplementary Fig. 7c, d). The defective substrate remodelling activities of the mutants can be due to several factors: the lower activation of Hsc70, the involvement of G/FR in client binding and JD positioning, and the role of ZFLR in substrate transfer to Hsc70^8,31^.

**Fig. 6:**
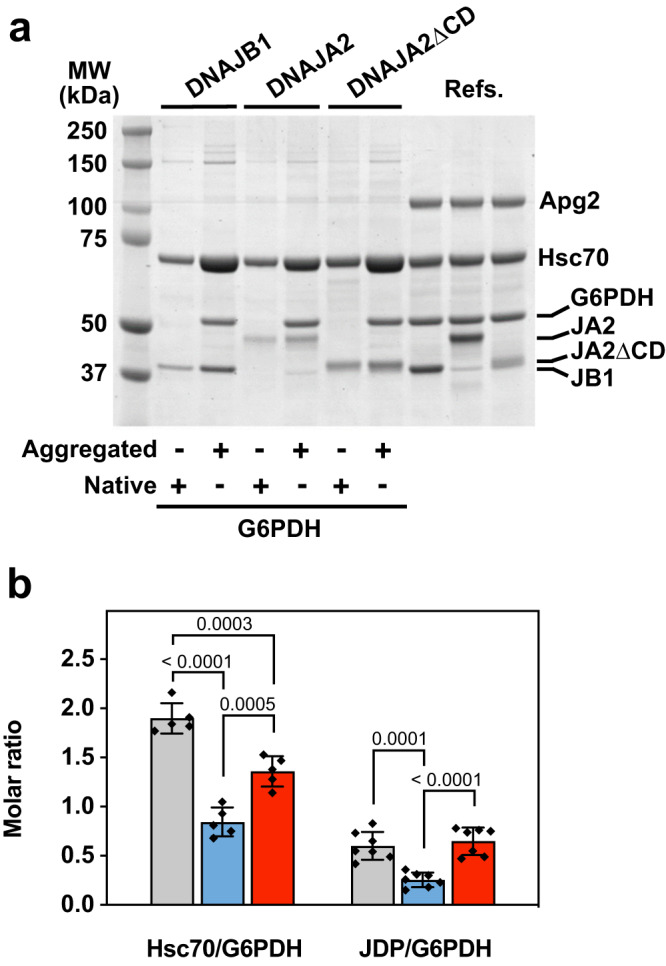
Hsc70 recruitment to protein aggregates depends on the identity of the J-domain protein used. **a** Interaction of 2 μM Hsc70 with native (−) or aggregated (+) G6PDH (0.4 μM) in the presence of different J-domain proteins (1 μM) and 0.4 μM Apg2. Reference lanes include 1 μg of each protein. Left, molecular weight markers (MW). **b** Hsc70/G6PDH and J-domain proteins/G6PDH molar ratios estimated in the pellets of samples containing DNAJB1 (grey), DNAJA2ΔCD (red) or DNAJA2wt (blue). The estimation was carried out quantifying band intensities from panel A. Data are mean ± SD of five (Hsc70/G6PDH) or seven (JDP/G6PDH) independent experiments. Significance was evaluated using a two-tailed, one-way analysis of variance (ANOVA) and Tukey´s multiple comparison, and the *p* values are shown in (**b**).

It is noteworthy the behaviour of DNAJA2ΔCD, which performed better than the wt protein with the two aggregated substrates both in the presence and absence of Apg2 (Fig. 5d, e). The disaggregation reaction is a sequential process in which JPDs bind first the aggregate and seek out Hsc70, transferring it to the aggregate surface where it extracts unfolded client molecules for their subsequent refolding. This has been demonstrated for the metazoan disaggregase both in vitro^32^ and in vivo^33^, using DNAJB1 instead of DNAJA2. Therefore, we used DNAJB1 as a reference in aggregate binding and disaggregation/refolding experiments. Reactivation of aggregates of luciferase (Fig. 5d) and G6PDH (Fig. 5e) by DNAJA2ΔCD ranked between those of DNAJA2wt and DNAJB1. The same difference between the disaggregating/refolding activities of these class A and B JDPs has been reported using luciferase aggregates^13^. Our finding suggests that CD removal might enhance productive interaction of Hsc70 with aggregated substrates. This suggestion was proved by measuring binding of each chaperone to aggregated G6PDH. Similar to what was previously found for DNAJB1^32^, DNAJA2 recruited Hsc70 to the aggregate surface, an activity that almost doubled in the presence of Apg2 (Supplementary Fig. 8). Interestingly, under our experimental conditions, the amount of aggregate-bound DNAJA2 was reduced around two-fold compared to DNAJB1, which resulted in a similar decrease in the JDP-dependent recruitment of Hsc70 to the aggregate surface (Fig. 6) and reactivation rates (Fig. 5d, e). This suggests that the limiting step of the association reaction, namely binding to the aggregate, explains the different Hsc70 recruiting and reactivation efficiencies. Removal of the DNAJA2 CD improved the interaction of the cochaperone with the aggregates, which increased the amount of Hsc70 being recruited (Fig. 6) and hence the reactivation rate (Fig. 5e). Therefore, the CD regulates the interaction of DNAJA2 with unfolded or aggregated substrates, favouring the former and hampering the later.

We then analysed the recovery of unfolded luciferase bound to Hsc70 or Apg2 assisted by DNAJA2wt, DNAJA2ΔC and DNAJB1 (Fig. 5f). This was accomplished by denaturing luciferase in the presence of Hsc70 (2 μM) or Apg2 (0.4 μM), which abolished substrate aggregation (Fig. 5b, inset). The refolding activity was assayed after addition of the other components of the Hsc70 system (Apg2/JDP or Hsc70/JDP). As found for the disaggregating/refolding activity, the folding activity of DNAJA2ΔCD was between those of DNAJA2wt and DNAJB1, i.e., significantly stronger than that of DNAJA2wt (Fig. 5f). Our finding suggests that besides being essential for the holding activity, the CD also regulates the interaction of the cochaperone with Hsc70 that leads to a productive substrate remodelling. It is worth mentioning the significantly higher refolding efficiency of the complete chaperone system (Hsc70/JDP/Apg2) when denaturation took place in the presence of DNAJA2 (78 ± 4%) as compared with Hsc70 (18 ± 3 %) or Apg2 (6 ± 2 %) alone (Fig. 5f). This indicates that the chaperone-bound conformation of luciferase might be distinct and/or remodelled differently depending on which protein protects it against aggregation, explaining why the same ternary chaperone mixture results in significantly distinct refolding yields.

### Modulation of DNAJA2 oligomerisation state by substrates and Hsc70

**Fig. 7:**
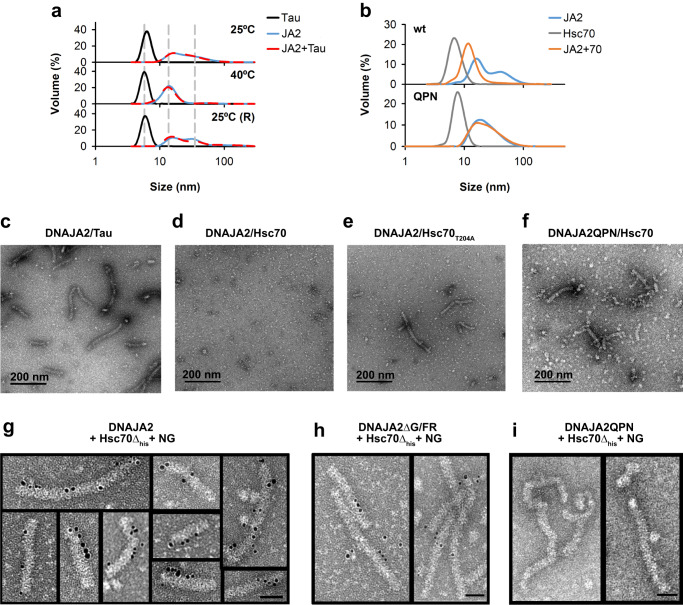
Modulation of DNAJA2 self-association by Tau and Hsc70. **a** Effect of tau K18 P301L on the temperature-dependence of the association equilibrium of DNAJA2, measured by DLS experiments in refolding buffer (20 mM Hepes-KOH pH 7.6, 50 mM KCl, 5 mM MgCl_2_ and 2 mM DTT). Volume against size distribution of 30 µM DNAJA2 recorded at 25 °C (top panel) or after incubating the sample 1 h at 40 °C (middle panel) in the absence (blue) or presence (dashed red) of 30 µM tau K18 P301L. The reversibility of the association reaction was analysed after an o/n incubation at 25 °C (bottom panel). A sample containing 30 µM tau K18 (black) was also analysed under the same conditions. **b** DLS of 30 µM DNAJA2wt (top panel) or DNAJA2QPN (bottom panel) after a 10 min incubation with (orange) or without (blue) 7 µM Hsc70Δlid in refolding buffer containing 3 mM ATP. As a control, a sample containing 7 µM Hsc70Δlid (grey) was analysed under the same conditions. Negative staining EM images of DNAJA2wt (15 µM) in the presence of equimolar amounts of (**c**) tau, (**d**) 7 µM Hsc70wt or (**e**) Hsc70 T204A, and of DNAJA2QPN in the presence of Hsc70wt (**f**). Samples were incubated 15 min at 25 °C in refolding buffer containing 2 mM ATP before staining. Negative staining images of mixtures of 15 µM DNAJA2wt (**g**), DNAJA2ΔG/FR (**h**), or DNAJA2QPN (**i**) and 7 µM His-tagged-Hsc70Δlid that were incubated 90 s at 4 °C. After the incubation, samples were treated with 5 nm Ni-NTA-Nanogold (NG) beads, washed and imaged. Bars in (**g**–**i**) indicate 100 Å. The images shown here are representative as they were observed in three different protein preparations.

The next question we wanted to address is whether the interaction of DNAJA2 with natural partners as unfolded substrates and Hsc70, modulates the oligomerisation state of the cochaperone. A partner-mediated change in DnaJ2A oligomerisation state would be expected if they interact with cochaperone domains that are involved in oligomer stability, and thus are able to outcompete these interdomain interactions. Tau K18 P301L was used to study the effect of the substrate, since its interaction with DNAJA2 has been characterised by NMR^27,28^. DLS experiments showed that equimolar amounts of monomeric tau changed neither the association equilibrium of DNAJA2 nor the reversible temperature-induced oligomer disassembly/reassembly (Fig. 7a). The negligible effect of tau was supported by EM images that displayed the characteristic tubular structures in the presence of equimolar amounts of this substrate (Fig. 7c). EM also showed that incubation of DNAJA2 with Hsc70 and ATP (2 mM) induced oligomer dissociation (Fig. 7d), suggesting that the chaperone disrupts the interdomain interactions that stabilise the oligomer. In contrast, when DNAJA2 was incubated under the same experimental conditions (15 min, 25 °C) with Hsc70 T204A, a chaperone variant that binds nucleotide but displays a 10-fold lower ATPase rate^34^, oligomer dissociation was considerably slowed down (Fig. 7e). Whereas large assemblies were not detected with Hsc70wt, they were still clearly visible in the presence of the ATPase defective Hsc70 variant. This mutant was used instead of completely inactive ones because its ATP-induced conformational rearrangement and response to JDPs are similar to those of the wt protein^35^. To prove the accessibility of the JD to Hsc70 in the tubular structure, we used the DNAJA2QPN variant which has the conserved J-domain HPD motif replaced by QPN. The same mutation in other JDPs abolishes their interaction with Hsc70^8,30^. This mutant was also able to form temperature-sensitive (Supplementary Fig. 9), elongated oligomeric structures that, in contrast to the wt protein, were not dissociated by the chaperone (Fig. 7f). Binding of Hsc70 to DNAJA2wt and DNAJA2ΔG/FR oligomers was characterised with a His-tagged, Hsc70Δlid variant that lacks the region (last 106 residues) responsible for chaperone oligomerisation. This variant interacts with the JDP auxilin and is functional in client remodelling^36^.

Incubation of this mutant with DNAJA2wt or DNAJA2ΔG/FR at low temperature (4 °C) for 90 s in the presence of 2 mM ATP allowed Hsc70 binding, which was monitored with Ni-coated beads that specifically label Hsc70 and were easily observed due to their strong electron density. EM images demonstrated that several Hsc70 molecules can bind to the same assembly of DNAJA2wt and DNAJA2ΔG/FR (Fig. 7g, h), and therefore that the JDs can search for and interact with Hsc70 within these oligomers. As expected, no binding was detected with the DNAJA2QPN variant (Fig. 7i and Supplementary Fig. 9). As Hsc70Δlid does not oligomerise, it was also employed to follow by DLS chaperone-mediated dissociation of large DNAJA2wt assemblies in the presence of ATP (Fig. 7b, top panel). In accordance with EM data, Hsc70-induced disassembly was not observed for the DNAJA2QPN variant (Fig. 7b, bottom panel). These findings strongly indicate that JDs are accessible to Hsc70 within the tubular structure of DNAJA2wt and point to the ATP-driven conformational cycle of Hsc70 as one of the factors responsible for assembly dissociation.

## Discussion

We analysed herein the ability of the class A human JDP DNAJA2 to self-assemble into dynamic, ordered oligomeric tubular structures, and the protein domains involved in oligomer stabilization. Our data indicate that a multivalent set of interactions are involved in oligomer formation and stabilisation, and that they include structured domains (JD, ZFLR and CTDI) and the disordered CD. Since JDPs have been implicated in different processes such as prevention of protein aggregation and assistance to Hsp70 in the remodelling of unfolded/aggregated substrates^37^, we also propose a functional role for the different oligomerisation states of DNAJA2.

Homo- and heterotypic, intermolecular interactions between different protein domains of adjacent DNAJA2 dimers stabilised the ordered, albeit flexible, oligomeric assembly. The tubular structure derived from cryoEM is composed of filaments formed by DNAJA2 dimers, which through lateral interactions adopt a helical arrangement with 5 dimers per disk (Fig. 3a). Docking of an atomic model of DNAJA2 into the experimental volume clearly pointed to two regions responsible for oligomer formation. Dimer-dimer interaction is procured by a specific region of CTDI (around residues L_135_ and Q_136,_ Fig. 4b) that generates unstable filaments (Supplementary Fig. 3f), which are stabilized by lateral contacts between filaments through the ZFLRs (Fig. 4c). This model is supported by mutational studies of these two regions, which were shown to impair formation of large assemblies (Fig. 1e, f and Supplementary Fig. 3e, f), although DNAJA2ΔZFLR still showed a small population of unstable filaments. Despite the fact that neither the CD nor JD were visible in the atomic model of the DNAJA2 oligomer, deletion of these domains also impaired DNAJA2 self-assembly, as deduced from DLS (Fig. 1d, e), crosslinking (Fig. 1f) and EM (Supplementary Fig. 3c, d). This finding could be explained considering the length and flexibility of the CD (52 residues predicted to be intrinsically disordered) and of the G/FR (unstructured region of 34 amino acids) that connects the JD to the DNAJA2 main body (Supplementary Fig. 1a). These two properties could allow both domains to reach any part of the DNAJA2 structure and thus influence oligomer formation. The crosslinks detected between CDs and both JDs and CTDI(ZFLR)s, and between JDs and CDs and CTDI(ZFLR)s would support this interpretation (see Fig. 3d, e and Supplementary Table 3).

The question that remains unanswered is what functional advantage the different oligomerisation states of DNAJA2 could have. This cochaperone has been described as a potent suppressor of tau seeding and intracellular amyloid formation, its levels being increased in neurons of Alzheimer disease patients^28^. Our data indicate that oligomers display a weaker holding activity than dimers, and thus they could function as a reservoir of dimeric species with a potentiated ability to avoid substrate aggregation. This seems reasonable due to the involvement of the client binding sites in oligomer stabilisation, as suggested by EM and mutational studies. Recent NMR data using a monomeric version of Ydj1, a DNAJA2 homologue from *Saccharomyces cerevisiae* that contains both CTDs and the ZFLR, revealed that a tau fragment similar to that employed in this study interacts mainly with the CTDI^27^. The involvement of this domain in contacts with adjacent JDs and CTDIs at the core of the DNAJA2 assembly, explains why oligomer dissociation enhances the protein-holding activity. Furthermore, the sensitivity of the association equilibrium to salt concentration and temperature suggests that under stress conditions, the oligomer could dissociate into dimers with fully exposed substrate binding sites that could complex unfolded clients with higher efficiency. A similar dissociation-induced activation of the holding activity was observed for small Hsps, which also assemble into polydisperse, large oligomers stabilised by multiple weak interactions between different protein domains^38^. These oligomers are sensitive to environmental changes, such as an increase in temperature, which shift the association equilibrium towards smaller species with higher holding activity due to exposure of substrate-binding sites that are sequestered in the oligomers^29^. Similarly, DNAJA2 oligomers could sense environmental changes and dissociate in harsh conditions. It is noteworthy the enhancement of the holding activity observed upon deletion of the JD. This functionally important domain, essential for the interaction with Hsc70, besides contacting adjacent CTDIs also interacts with CDs in the tubular assembly, and therefore could shield, at least partially, these substrate binding sites. Truncation of the JD dissociates the oligomer and would expose these sites, explaining the stronger holding activity of DNAJA2ΔJD. A regulatory interaction between the JD and the CTD has also been described in DnaJB8, and has been proposed to mediate Hsc70 recruitment to substrate-bound cochaperone^15^.

Deletion of the CD abrogates the holding activity of the protein, despite the presence of the CTDs. This might be expected if reduction in the substrate binding sites and oligomer dissociation upon elimination of this domain would negatively affect the holding activity of the protein, likely affecting its avidity for client proteins. The interactions between CDs and CTDs observed by XL-MS could also modulate the conformation of the client binding regions (CTDs), as found for a fragment of Ydj1 containing the CTDs and the CD^39^, and therefore the holding activity of the protein. The DNAJA2ΔCD variant forms dimers in solution, as evidenced by DLS (Fig. 1d), cross-linking (Fig. 1f), and by its ability to collaborate with Hsc70 in substrate refolding better than the wt protein (Fig. 5e, f). If deletion of the CD were to destabilize the dimer, severe dysfunctions in the chaperone activity of the Hsc70 system would be expected, as disruption of the dimerization motifs of class A and B JDPs drastically compromises the chaperone functions^40,41^. In contrast, we show that deletion of the CD improves collaboration of DNAJA2 with Hsc70. Although the structural basis for this behaviour is not straightforward to elucidate due to the flexibility of these domains, the CD could sterically difficult the interaction of Hsc70 with the JD. Crosslinks between these two domains (Supplementary Table 3) support this interpretation and suggest that their interaction, among others discussed above, could regulate DNAJA2 function.

**Fig. 8:**
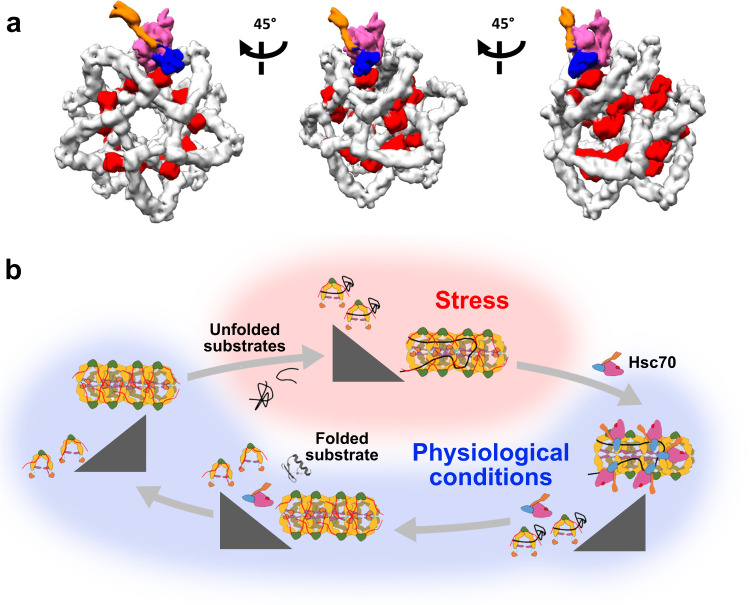
Proposed model for the interaction of DNAJA2 with client proteins and with Hsc70. **a** The dimensions and shape of the Hsc70(ATP) state are complementary to the grooves of the tubular, helical structure of the DNAJA2 assembly. This is neccessary for the intial interaction of this Hsc70 conformation with the JD of the cochaperone, which is accessible to Hsc70 in the tubular assembly. **b** Model proposed to explain the benefits that the association equilibrium of DNAJA2 could have in the holding and substrate remodelling activities of the Hsc70 system. Under heat shock (stress) conditions, DNAJA2 oligomers dissociate into smaller species with the client binding sites exposed to complex unfolded client proteins, and therefore with an enhanced holding activity. The height within the triangle reflects the abundance of the corresponding oligomerization states. Once the physiological conditions are restored the cochaperone-substrate protein complex might associate into large oligomers. The array of nearby JDs in the oligomer could act as a scaffold, allowing simultaneous binding of several Hsc70 molecules to the chaperone-bound, unfolded client for an efficient substrate remodelling. The conformational cycle of bound Hsc70 molecules, driven by ATP hydrolysis, that is necessary for substrate remodelling would dissociate the DNAJA2 assembly, releasing free DNAJA2 dimers that could reassociate to start the cycle.

Our data also indicate that tau does not perturb the reversible temperature-induced dissociation of DNAJA2 and thus that once the stress conditions disappear, DNAJA2 can assemble into tubular structures in the presence of this substrate. This suggests that tau neither outcompetes the intermolecular interactions that stabilise the oligomeric assembly nor impedes the association of the temperature-induced dimers upon cooling of the sample. However, given the plethora of client proteins, we cannot discard that other unfolded substrates could induce oligomer dissociation. The DNAJA2 assembly could function as a scaffold with an array of closely spaced JDs to promote simultaneous binding of several Hsc70 molecules to DNAJA2-bound substrates. Accessibility of the JDs to Hsc70 in the tubular structure is clearly observed by EM (Fig. 7g–i), which shows that a DNAJA2 oligomer can bind several Hsc70 molecules using the JDs of different DNAJA2 molecules. The structural complementarity between Hsc70(ATP), the chaperone conformation that binds DNAJA2, and the groove of the cochaperone oligomer might facilitate initial interaction of Hsc70 with the JD of DNAJA2 (Fig. 8a). The conformational rearrangement of JD-bound Hsc70 during its ATPase, functional cycle^35^ would trigger dissociation of the DNAJA2 assembly. Once the closely DNAJA2-bound Hsc70 molecules are transferred to the unfolded substrate, cooperative substrate remodelling would explain the higher recovery yield observed when the substrate is protected from aggregation by DNAJA2 as compared to Hsc70 and Apg2.

In summary, we propose a model in which the self-association equilibrium of DNAJA2 would modulate its interaction with unfolded substrates and with Hsc70 (Fig. 8b). DNAJA2 assembles into ordered, tubular oligomers stabilized by interactions between domains responsible for Hsc70 (JD) and client protein (CTDs) binding. In harsh conditions, such as heat shock temperatures, DNAJA2 assemblies dissociate into dimers with fully exposed substrate binding sites and therefore a stronger holding activity. Once proteotoxic stress conditions cease, the dimer-substrate complexes can reassemble into oligomers with an array of closely spaced JDs that allow binding of several Hsc70 molecules that can be transferred to an unfolded substrate molecule. This would facilitate cooperative substrate processing and a high refolding efficiency. Cochaperone dissociation, triggered by Hsc70, could facilitate the conformational cycle of Hsc70 required for the refolding of client proteins. This model is based on our in vitro findings, and remains to be carefully assessed in vivo to elucidate its cellular relevance.

## Methods

### Cloning, expression and purification of proteins

The cDNAs of Apg2 (HSPH2), Hsc70 (HSPA8), DNAJB1 and DNAJA2 were obtained from Addgene and cloned into a pE-SUMO vector (LifeSensors, Malvern, USA). The deletion mutants DNAJA2ΔJD, DNAJA2ΔG/FR, DNAJA2ΔZFLR, DNAJA2ΔCD, and CD carrying a deletion from residue 1 to 74, 77 to 101, 141 to 209, 361 to 412, or 1–361, respectively, were cloned by fusing two PCR fragments corresponding to the upstream and downstream sequences of these protein segments. DNAJA2LQ was generated by restriction digest-based mutagenesis and DNAJA2QPN was produced by GeneScript (Rijswijk, Netherlands). All mutants were verified by sequencing. Recombinant chaperones containing a tag with 6xHis and SUMO fused to the N-terminus were expressed in BL21 Rosetta or BL21-CodonPlus (DE3)-RIPL cells and purified as described^32^. Tau K18 C291A, C322A, P301L (K18 P301L) was cloned into a pNG2 vector, expressed in *E. coli* BL21 (DE3) cells and purified as described^42^. Purification of human 6x His-tagged Hsc70Δlid was performed as reported^36^, with an extra purification step. Briefly, after expression, the lysate was loaded onto a HisTrap column and the collected fractions were subjected to anion exchange on Q-Sepharose (pH 8.0), cation exchange on S-Sepharose (pH 6.5), and gel exclusion on Superdex 200.

### Dynamic light scattering

Samples contained 30 μM DNAJA2 in buffer composed of 50 mM KCl, 2 mM DTT and 20 mM Hepes-KOH pH 7.6. The effect of ionic strength (300 mM NaCl), 5% 1,6-hexanediol and temperature (40 °C) on the self-association of DNAJA2wt was analysed in a Nano-S Zetasizer (Malvern Panalytical) with a 173° backscatter detector and using disposable cuvettes (10 mm light path, Eppendorf). In all measurements, 30 accumulations were collected, each of 10–16 measurements. The effect of Hsc70Δlid (7 μM) was studied in samples containing 30 μM DNAJA2 and 3 mM ATP. After incubating them 10 min in refolding buffer (50 mM KCl, 2 mM DTT, 5 mM MgCl_2_, 20 mM Hepes-KOH pH 7.6) at 25 °C, samples were measured at 20 °C. Data were analysed with Zetasizer family software (v7.13, Malvern Panalytical), taking the viscosity and refractive index of the buffer into account, and the results were represented as volume-based distributions. DLS measurements were performed in at least two distinct protein batches of the different protein species.

### ATPase assay

The ATPase activity of the samples was measured as described^43^. These experiments were performed in a Synergy HXT plate reader (BioTek) at 30 °C in 40 mM Hepes pH 7.6, 50 mM KCl, 5 mM (CH_3_COO)_2_Mg and 2 mM DTT buffer. Protein concentrations were 2 μM Hsc70, 0.4 μM Apg2 and increasing concentrations (0–5 μM) of J-domain proteins. The ATPase-regeneration system (0.3 mM NADH, 3 mM PEP, 20 ng/mL PK, 0.017 mg/mL lactate dehydrogenase (LDH) and 2 mM ATP) was added to initiate the reaction. ATP consumption rates (μmol ATP/min) were calculated from slopes of the A_340_ decay curves that presented a linear absorbance decline using the extinction coefficient of NADH (${ {\mathcal E} }_{340}$ 6220/M/cm).

### Holding activity

The aggregation of tau K18 P301L was monitored recording the light scattered at 350 nm for 16 h at 37 °C. This truncated variant of tau formed from the aggregation-prone repeats of the microtubule-binding domain (residues 244–372) aggregates faster than the full-length protein^44^. This construct also contains the missense mutation P301L that has been linked to familial frontotemporal dementia^45^ and further favours aggregation. Samples were prepared by adding 6 μM of the different JDPs to mixtures of 20 μM tau K18 P301L and equimolar amounts of heparin (MP Biomedicals) in buffer containing 25 mM Tris-HCl pH 7.5, 200 mM NaCl and 2 mM DTT. The lag phase of tau K18 P301L aggregation was determined as the cross-over between the lag-phase and the growth-phase. Recombinant luciferase from Photinus pyralis (Sigma-Aldrich) (0.125 μM) was incubated for 40 min at 42 °C in 40 mM Hepes-KOH pH 7.6, 50 mM KCl, 5 mM MgCl_2_ and 2 mM DTT in the absence or presence of different DNAJA2 variants (1 μM). The insulin aggregation assay was carried out in 40 mM Hepes/KOH pH 7.5. Samples contained 45 μM insulin and increasing concentrations of DNAJA2wt (0, 1, 2, 4, 7 and 10 μM) in conditions that favour oligomers (18 °C/0 mM NaCl) or dimers (18 °C/300 mM NaCl or 42 °C/0 mM NaCl). They were incubated 30 min at the respective conditions and the aggregation reaction was started by the addition of freshly prepared DTT to a final concentration of 15 μM. Aggregation was monitored at 400 nm in a Synergy HTX plate reader (BioTeK) using white 96-well plates with flat bottoms (Sarstedt) in a final volume of 120 μL. Data are mean ± SD of at least three independent experiments.

### Refolding of unfolded luciferase

Luciferase (0.125 μM) was denatured for 40 min at 42 °C in 40 mM Hepes-KOH pH 7.6, 50 mM KCl, 5 mM MgCl_2_ and 2 mM DTT containing 2 mM ATP, 3 mM PEP and 20 ng/mL PK in the absence or presence of different chaperones (2 μM Hsc70, 1 μM J-domain proteins or 0.4 μM Apg2). Then, samples were incubated at 30 °C for 5 min before adding different chaperone combinations at concentrations indicated above, so that each sample would end up with the same ternary (Hsc70, JDPs and Apg2) chaperone combination. The recovery of luciferase (0.1 μM) was measured after 2 h at 30 °C by adding 5 μL of each sample to 50 μL luciferin (Promega, E1500) and recording the luminescence in a Synergy HTX plate reader (BioTeK) using white 96-well plates with flat bottoms (Sarstedt). The activity of non-denatured luciferase in the presence of the ternary chaperone mixture was set as 100%, and 0% activity corresponded to chaperone-untreated luciferase.

### Disaggregating activity

Recombinant luciferase from *P. pyralis* (Sigma-Aldrich) was aggregated and reactivated by the Hsc70 system as in previous works^32^, as it was Glucose-6-phosphate dehydrogenase (G6PDH) from *Leuconostoc mesenteroides* (Worthington)^46^. Reactivation percentages were calculated considering the activity of the aggregated and native (in the presence of chaperones) proteins as 0% and 100%, respectively.

### Chaperone binding to G6PDH aggregates

The interaction of chaperones with G6PDH aggregates was followed with a co-sedimentation assay previously described^32^. The amount of aggregate-bound chaperones was estimated relative to that of aggregated G6PDH by measuring the intensity of bands using a gel scanner G-800 and the Quantity One software (Bio-Rad). Each data point is the average of at least three independent experiments and was estimated by subtracting the amount of protein found in pellets of control experiments, containing native G6PDH, which were carried out in parallel.

### Crosslinking and mass spectrometry (XL-MS) analysis

50 μg of DNAJA2 and DNAJA2ΔG/FR were first subjected to chemical crosslinking by incubation with 2 mM BS3 in 50 mM Na-Hepes pH 7.0, 20 mM NaCl for 40 min at RT with 600 rpm shaking. The reactions were quenched for 15 min at RT by adding 50 mM Tris-HCl pH 7.0. Purified BS3-crosslinked DNAJA2 or DNAJA2ΔG/FR were incubated in Laemmli sample buffer (0.02% [w/v] bromophenol blue, 2% [w/v] SDS, 10% [v/v] glycerol, 60 mM Tris-HCl pH 6.8) for 5 min at 96 °C and loaded onto a 12% polyacrylamide gel. The gel was stained with Quick Coomassie (Generon), showing a set of bands with different molecular weights, from which only the largest, which did not enter the resolving part of the gel and were compatible with the large assemblies observed by EM, were excised and subjected to automated reduction, alkylation with iodoacetamide and trypsin digestion in a Proteineer DP robot (Bruker Daltonics). The resulting peptide mixture was speed-vac dried and re-dissolved in 0.1% (v/v) formic acid. Liquid chromatography–mass spectrometry (LC-MS/MS) analysis was carried out using a nano-LC Ultra HPLC (Eksigent, Framingham, MA) coupled online to a 5600 triple TOF mass spectrometer (AB Sciex, Framingham, MA) through a nanospray III ion source (AB Sciex) equipped with a fused silica PicoTip emitter (10 μm × 12 cm; New Objective, Woburn, MA). Peptides were fractionated at a flow rate of 0.250 mL/min at 50 °C under gradient elution conditions. The ion source was operated in positive ionization mode at 150 °C with a potential difference of 2300 V.

For peptide identification, raw MS data were searched against a custom-made database containing the amino-acid sequence of human DnaJA2 or DnaJA2∆G/FR. The MS/MS ion search was performed with Stavros/MeroX 2.0^47^ using its quadratic mode of analysis. BS3 was selected as crosslinker with specificity for Lys and protein N-terminus at site 1 and Lys, Ser, Thr, Tyr and protein N-terminus at site 2. Intra-peptideal and dead-end crosslinks by reaction with H_2_O were considered. Trypsin was selected as enzyme allowing 3 missed cleavages. Carbamidomethylation (Cys) and oxidation (Met) were set as fixed and variable modifications, respectively. Analysis was performed with MS and MS/MS tolerances of 10 and 20 ppm and with lower and upper mass limits of 500 and 7000 Da, respectively. For peptide matching, only precursors with a charge state >1+ and at least three fragments of the a, b or y series per peptide were considered. For scoring, the slowest and most precise method was chosen, excluding internal linear ions. The decoy database was generated by shuffling sequences but keeping the protease sites. A prescore (% intensity) of 10 was applied and peptide identifications were filtered using a XlinkX score > 30 and a false discovery rate (FDR) < 5%. All the MS2 spectra of the resulting peptides were manually revised. XL-MS data have been deposited to the ProteomeXchange Consortium via the PRIDE partner repository with the dataset identifier PXD043837.

In addition, samples were crosslinked with glutaraldehyde^48^. Samples containing 2 μM DNAJA2 were incubated without or with 0.005% (v/v) glutaraldehyde for 15 min at 4 °C in 20 mM Hepes pH 7.6, 50 mM KCl, 5 mM MgCl_2_ and 2 mM DTT. The reaction was stopped by the addition of 100 mM Tris-HCl pH 7.5. Finally, SDS-loading buffer was added to the samples that were heated at 90 °C for 10 min, resolved by SDS-PAGE (10%) and stained with Coomassie Brilliant Blue.

### Negative staining electron microscopy

Aliquots (5 μL) of the different samples (DNAJA2wt, DNAJA2 mutants, DNAJA2wt/Hsc70 complexes, nanogold labelling of Hsc70) were applied onto glow-discharged carbon-coated 300-mesh copper grids and incubated for 1 min. Grids were negatively stained with 2% (*w*/*v*) uranyl acetate for 1 min and air-dried for 5 min. Images were taken using a JEOL JEM 1400 electron microscope operated at 120 kV and equipped with a CCD camera (4 K × 4 K TemCam-F416, TVIPS). Images were recorded at a 50,000 × nominal magnification with a sampling rate of 2.4 Å/px. The representative negative-stain EM images were observed in independent experiments with three different protein preparations.

### Nanogold labelling of Hsc70

The localization of His-tagged Hsc70Δlid was performed using 5 nm Ni-NTA-Nanogold (NG) beads (Nanoprobes). First, DNAJA2wt, DnJA2ΔG/FR and DNAJA2QPN (15 μM) were incubated 90 s with 6x His-tagged Hsc70Δlid in refolding buffer at 4 °C in the presence of 2 mM of ATP. Samples (5 μL) were applied onto glow-discharged carbon-coated 300-mesh grids and incubated 1 min. The excess liquid was removed using filter paper and the grids were incubated with 5 μL droplets of 1/5 diluted NG for 30 min at RT. Unbound NG was removed by incubation with droplets of 10 mM imidazole in 20 mM Tris, pH 7.6, 150 mM NaCl, for 1 min at RT and washed with water.

### Sample preparation for CryoEM

Oligomers of DNAJA2wt and DNAJA2ΔG/FR at 30 μM were stabilized using 2 mM BS3. First, maintenance of the size and shape of the oligomers after crosslinking was assessed using negative staining EM. Once this was confirmed, crosslinked samples were used for the CryoEM studies to prevent oligomer disassembly upon dilution to suitable concentrations for vitrification. Aliquots of 4 μL of the different samples were vitrified using a Vitrobot Mark IV (FEI) and were incubated onto Quantifoil R 2/2 300 mesh grids, blotted for 2 s at 22 °C and 95% humidity and plunged into liquid ethane.

### CryoEM data acquisition

The DNAJA2wt cryoEM grids were checked, and data from the best one acquired in a 200 kV FEI Talos Arctica equipped with a Falcon III direct electron detector at the Centro Nacional de Biotecnología (CNB) cryoEM facility. A total of 1482 movies were acquired at a nominal magnification of ×73000 (corresponding to a pixel size of 1.37 Å/pixel), with a defocus range of −1.2 to −3.4 μm. Movies were fractionated to 60 frames with an exposure time of 1 s and a dose rate of 61 e^−^/pixel/s The total accumulated dose was 32 e^−^/Å^2^ (0.53 e^−^/Å^2^/frame). DNAJA2ΔG/FR samples were first checked on a 200 kV FEI Talos Arctica at the CNB followed by data acquisition on a FEI Titan Krios electron microscope (Krios 1) operated at 300 kV, equipped with a Gatan Quantum K3 Summit direct electron detector mounted on a Gatan Bioquatum LS/967 energy filter at the European Synchrotron Radiation Facility (ESRF) in Grenoble. Data collection was carried out with a 130000x nominal magnification (yielding a pixel size of 1.053 Å/pixel) and a defocus range of −1.2 to −2.6 μm. A total of 10714 movies were recorded and each movie was fractionated to 40 frames with an exposure time of 5 s and a dose rate of 8.15 e^−^/pixel/s. The total accumulated electron dose was 36 e^−^/ Å^2^ (0.90 e^−^/Å^2^/frame). The representative cryoEM images shown in this study were observed in independent experiments with three different protein preparations.

### Image processing and three-dimensional reconstruction

Image processing of DNAJA2wt and DNAJA2ΔG/FR was performed following a similar workflow (Supplementary Fig. 5). All programs used for image processing to obtain the different 3D maps were implemented in the Scipion software platform^49^. First, the movies were aligned using MotionCor2^50^ and the outputs were subjected to CTF determination using Gctf^51^. DNAJA2wt oligomers observed in the images were segmented and treated as single particles that were manually collected using Xmipp3- manual picking^52^. The 49387 extracted particles were subjected to an initial 2D classification using CryoSPARC^53^. The best classes were subjected to several further rounds of 2D classification using Relion 2.0^54^, allowing better detection of “bad” particles, such as particles that were very close to each other and ice contamination. Some of the best 2D classes were used as a template to generate an initial model using RANSAC^55^, and a second initial model was made from a blob with the dimensions obtained from the 2D averages. This ab initio modelling, together with the filtering of the initial models at low resolution in each step of the refinement, prevents the introduction of any model bias in the 3D reconstruction process. Both initial models were low-pass filtered to 60 Å and used for a 3D classification of the particles selected in the best 2D classes without symmetry or handedness imposition. The 11,388 particles of the best class were used for a further 3D auto-refine using Relion 2.0 and yielded an oligomeric structure of DNAJA2wt at 12.7 Å with helical appearance and compatible with C5 and D5 symmetry. Imposition of D5 symmetry generated a better, higher resolution structure of the oligomeric assembly at 8.7 Å resolution.

For DNAJA2ΔG/FR, image processing was performed following similar steps while using the SPHIRE-CRYOLO automated particle picker^56^ and Relion-3^57^. After several rounds of 2D classification, the selected particles were 3D classified using either the final map of DNAJA2wt and/or one of the ab initio models (with similar results), filtered at 50 Å to prevent model bias, without imposing symmetry or handedness. The 47396 selected particles were used for C1 and D5 refinements to generate the final 3D maps at 9.2 and 6.9 Å resolution, respectively, as estimated by the gold standard Fourier shell correlation (FSC), which uses a cutoff value of 0.143. Since the helical structure of the oligomers was very evident, an attempt to apply helical symmetry to the DNAJA2ΔG/FR filaments was carried out with two of the maps obtained using the D5 symmetry. The analysis was performed using three consecutive rings of DNAJA2ΔG/FR and, despite a decrease in the resolution, an average helix pitch for each of the two structures was obtained. To calculate this, the program “xmipp_volume_find_symmetry” (included in Scipion) was used, which performs an exhaustive search for symmetry by cross-correlation in real space. The helical parameters obtained were applied to the volumes using “xmipp_transform_symmetrize” (included in Scipion) and the resulting maps were visualized with Chimera. Unfortunately, the high degree of heterogeneity in the particles did not generate a higher resolution than that obtained by applying D5 symmetry.

The final C1 3D map of DNAJA2ΔG/FR was used to localise the JDs. After the final refinement, particles were subjected to a 3D-Relion classification without alignment using a mask in the regions where the JDs are expected according to the DNAJA2ΔG/FR sequence. Particles belonging to the class with better resolution in the area of the JDs were subjected to cryosparc non-uniform (optimizing per particle defocus and per-group CTF parameters) and cryosparc local refinement protocols.

### Homology modelling, molecular dynamics simulations and docking

We resorted to homology modelling to generate an atomistic model of DNAJA2. The first step was to search for the best templates by scanning the query sequence against SWISS-MODEL^58^, Phyre2^59^ and I-TASSER^60^ servers. Then, the templates were aligned with the query protein considering the structure, and the structure-based alignment was used to generate the model using MODELLER^61^. Different models were generated and all of them were analysed by ERRAT^62^, Verify-3D^63^ and WHAT-IF^64^ to assess their quality. The best model was selected, energy minimized, and refined by MD simulations using OMMprotocol^65^ as a command line application to launch MD simulations with OpenMM^66^.

MD calculations were prepared for all-atom simulations: histidines predicted to be in a region with pH > 6.0 by H++ server^67^ were protonated, and the system was set up with xleap^68^. Explicit solvent was used and Na+ ions were added to attain charge neutrality. The model system was embedded with a distance between the protein and the box edge of 6 Å. AMBER ff14SB^69^ and TIP3P^70^ force fields were used for proteins and water, respectively. Parameters for the ZFLRs were taken from the literature^71^.

MD simulations were performed using OpenMM^66^ with the following conditions: A cutoff of 1 nm was used for short range electrostatics and Van der Waals interactions. Long range electrostatic interactions were calculated with the Particle-Mesh Ewald method (PME), using periodic boundary conditions^72^. Bonds involving hydrogen atoms were constrained using the SHAKE algorithm^73^. A time step of 1 fs was used to integrate the equation of motion with a Langevin integrator^74^. Constant temperature and pressure were achieved by coupling the systems to a Monte Carlo barostat at 1.01325 bar^75^. Model systems were energy minimized progressively before starting the MD simulations. Then, the temperature was increased from 100 to 300 K in order to achieve the thermalisation of water molecules and side chains, and finally MD simulations of 100 ns were carried out and further analysed. The UCSF Chimera package was used for visualization and molecular graphics^76^.

The best model of the DNAJA2 dimer was shaped into the experimental map using the UCSF Chimera software and Coot^77^, and subsequently refined with a standard procedure using PHENIX 1.20-4459^78^ or REFMAC^79^ included in the CCP-EM software platform^80^. Several rounds of real-space refinement were performed until a stable final model was obtained for validation using Molprobity^81^ (Supplementary Table 2).

### Statistical analysis and reproducibility

Statistical analysis was performed in GraphPad Prism 9.3.1 using a two-tailed, one-way analysis of variance (ANOVA) and Tukey´s multiple comparison test. The *p* values obtained with this analysis are shown in the corresponding Figures. A value of *p* < 0.05 was considered statistically significant. The representative negative-stain EM and CryoEM micrographs shown in this study were observed in independent experiments with three different protein preparations.

### Reporting summary

Further information on research design is available in the Nature Portfolio Reporting Summary linked to this article.

### Supplementary information

Supplementary Information

Reporting Summary

### Source data

Source Data

## Data Availability

Cryo-EM data have been deposited in the Electron Microscopy Data Bank under accession codes EMD-14729 for DNAJA2 wt and EMD-14706 for DNAJA2 ΔG/FR. The two maps obtained using D5 symmetry were also deposited under accession codes EMD-14727 and EMD-14736. The D5 DNAJA2ΔG/FR associated atomic model has been also deposited in the Protein Data Bank under accession code 7ZHS. The XL-MS data have been deposited in the ProteomeXchange Consortium via the PRIDE partner repository with the dataset identifier PXD043837. Source data underlying Figs. 1, 5, 6, 7, and Supplementary Figs. S6, S7, S8 and S9 are provided with this paper as a Source Data file. Source data are provided with this paper.
