# Author Correction: The self-association equilibrium of DNAJA2 regulates its interaction with unfolded substrate proteins and with Hsc70

**DOI:** 10.1038/s41467-023-44499-y

**Published:** 2024-01-02

**Authors:** Lorea Velasco-Carneros, Jorge Cuéllar, Leire Dublang, César Santiago, Jean-Didier Maréchal, Jaime Martín-Benito, Moisés Maestro, José Ángel Fernández-Higuero, Natalia Orozco, Fernando Moro, José María Valpuesta, Arturo Muga

**Affiliations:** 1 Biofisika Institute (CSIC, UPV/EHU), University of the Basque Country, 48940 Leioa, Spain; 2 Department of Biochemistry and Molecular Biology, Faculty of Science and Technology, University of the Basque Country (UPV/EHU), 48940 Leioa, Spain; 3 Department of Macromolecular Structure, National Centre for Biotechnology (CNB-CSIC), 28049 Madrid, Spain; 4 Insilichem, Departament de Química, Universitat Autònoma de Barcelona, (UAB), 08193 Bellaterra (Barcelona), Spain

**Keywords:** Cryoelectron microscopy, Chaperones, Chaperones, Chaperones

Correction to: *Nature Communications* 10.1038/s41467-023-41150-8, published online 05 September 2023

In the original version of this Article, the Acknowledgements section incorrectly omitted one of the funding sources. Incorrect sentence ‘This research was supported by grants PID2019-111068GB-I00 (AEI/FEDER, UE) to A.M., PID2020-117752RB-I00 (AEI/FEDER, UE) to J.M.B., and PID2019-105872GB-I00 and PID2022-137175NB-I00 (AEI/FEDER, UE) to J.M.V. and J.C., and by the Basque Government (grant IT1201-19 to F.M.)’ has been now replaced with ‘This research was supported by grants PID2019-111068GB-I00 (AEI/FEDER, UE) to A.M., PID2020-117752RB-I00 (AEI/FEDER, UE) to J.M.B., and PID2019-105872GB-I00 and PID2022-137175NB-I00 (AEI/FEDER, UE) to J.M.V. and J.C., and by the Basque Government (grants IT1201-19 and IT1745-22) to F.M.)’. This has been corrected in both the PDF and HTML versions of the Article.

