# Supplementary material for: The self-association equilibrium of DNAJA2 regulates its interaction with unfolded substrate proteins and with Hsc70
Source: Nat Commun. 2023 Sep 5;14:5436. doi: 10.1038/s41467-023-41150-8 (PMC10480186; doi:10.1038/s41467-023-41150-8)
Supplement: Supplementary file 1 — Supplementary Information [file 41467_2023_41150_MOESM1_ESM.pdf]

SUPPLEMENTARY INFORMATION

The self-association equilibrium of DNAJA2 regulates its interaction with unfolded substrate proteins and with Hsc70

Lorea Velasco-Carneros*[1,2], Jorge Cuéllar*[3], Leire Dublang*[1,2], César Santiago[3], Jean-Didier Maréchal[4], Jaime Martín-Benito[3], Moisés Maestro[3], José Ángel Fernández-Higuero[1,2], Natalia Orozco[1], Fernando Moro[1,2], José María Valpuesta[&3], Arturo Muga[&1,2]

[1] Biofisika Institute (CSIC-UPV/EHU), University of the Basque Country, 48940, Leioa, Spain.

[2] Department of Biochemistry and Molecular Biology, Faculty of Science and Technology, University of the Basque Country (UPV/EHU), 48940, Leioa, Spain.

[3] Department of Macromolecular Structure, National Centre for Biotechnology (CNB-CSIC), 28049 Madrid, Spain.

[4] Insilichem, Departament de Química, Universitat Autònoma de Barcelona, (UAB), 08193 Bellaterra (Barcelona), Spain.

This PDF file includes:

Supplementary Figures 1 to 9 and Supplementary Tables 1 to 3.

SUPPLEMENTARY FIGURES

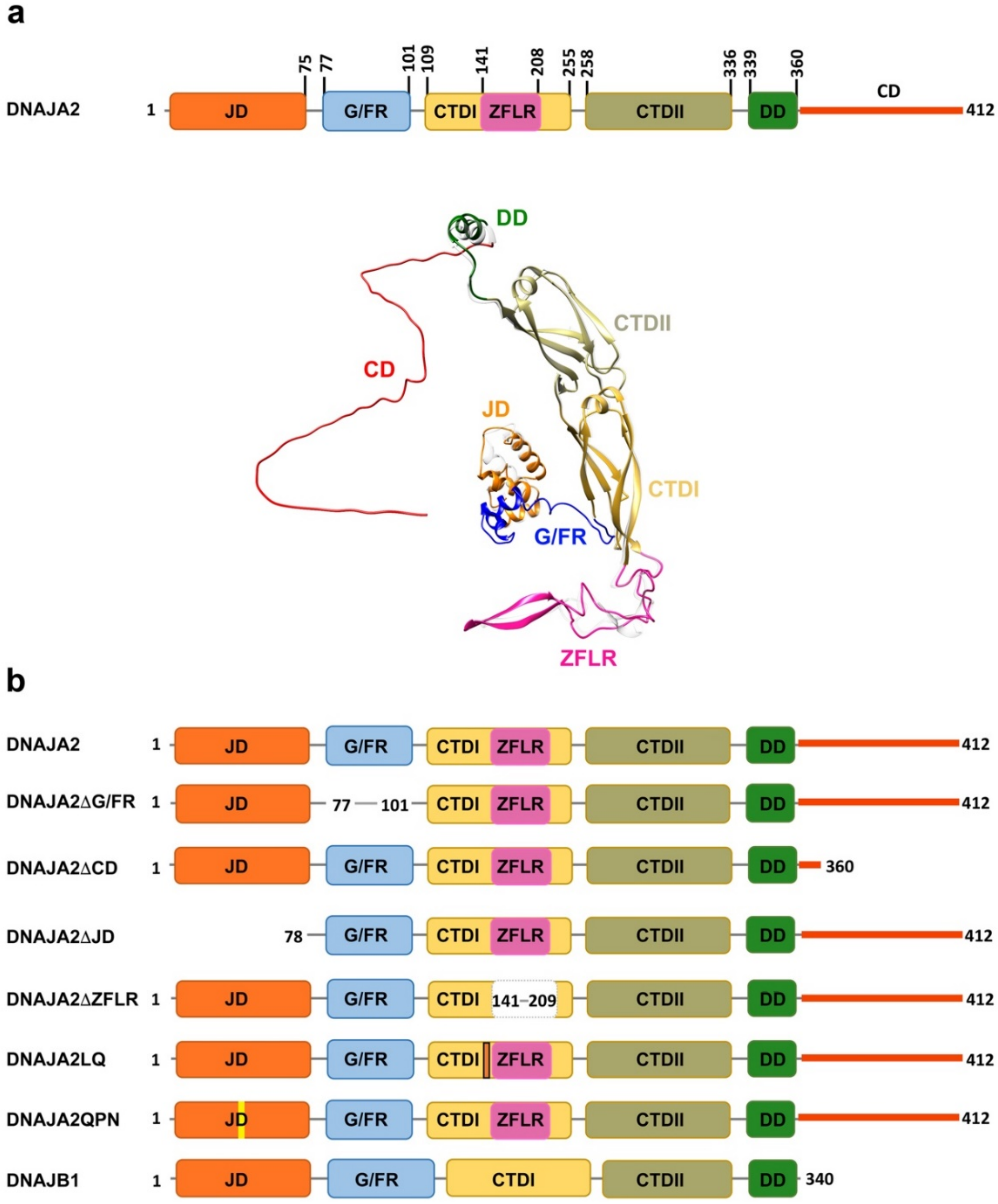

Supplementary Figure 1. Domain organization of DNAJA2 and the variants used in this study. (a) Superposition of the Alphafold model[1] (gray) and the homology model of the DNAJA2 monomer with the different domains: the J-domain (JD; orange), the glycine/phenylalanine-rich domain (G/FR; blue), the two homologous β sandwich domains (CTDI and CTDII; yellow and khaki), with a Zn2+ finger-like region (ZFLR; pink) inserted into the first one, the dimerization domain (DD; green). The model lacks the G/FR and CD because no templates were found for these intrinsically disordered regions. Alphafold models these domains (G/FR in blue and the CD in red) with low/very low confidence and are drawn only to note their length and flexibility. (b) Schematic representation of DNAJA2wt and the protein variants used in this study. The domain organization of DnaJB1, a dimeric class B Hsp40, is also shown.

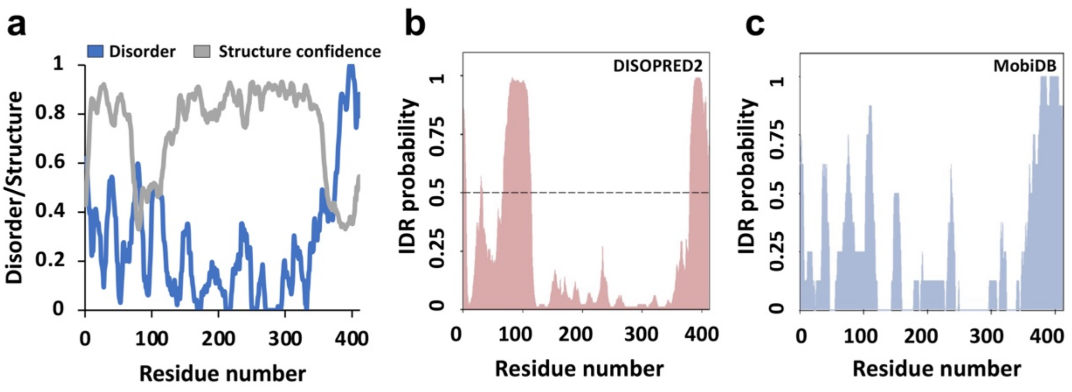

Supplementary Figure 2. Prediction of disordered regions in DNAJA2. (**a**) Metapredict analysis of the DNAJA2 sequence showing the predicted disorder (blue) and the AlphaFold-derived structural confidence score (grey). (**b**) Prediction of disordered regions by DISOPRED2 and (**c**) MobiDB. Plots show the position in the protein sequence against the probability of being disordered. The threshold for DISOPRED2 is indicated with a dashed line at 0.5 and the threshold for MobiDB consists of a minimum of 20 consecutive residues with a probability of being disordered greater than 0.5. The last 48 residues of DNAJA2 form the only region that meets this criterion.

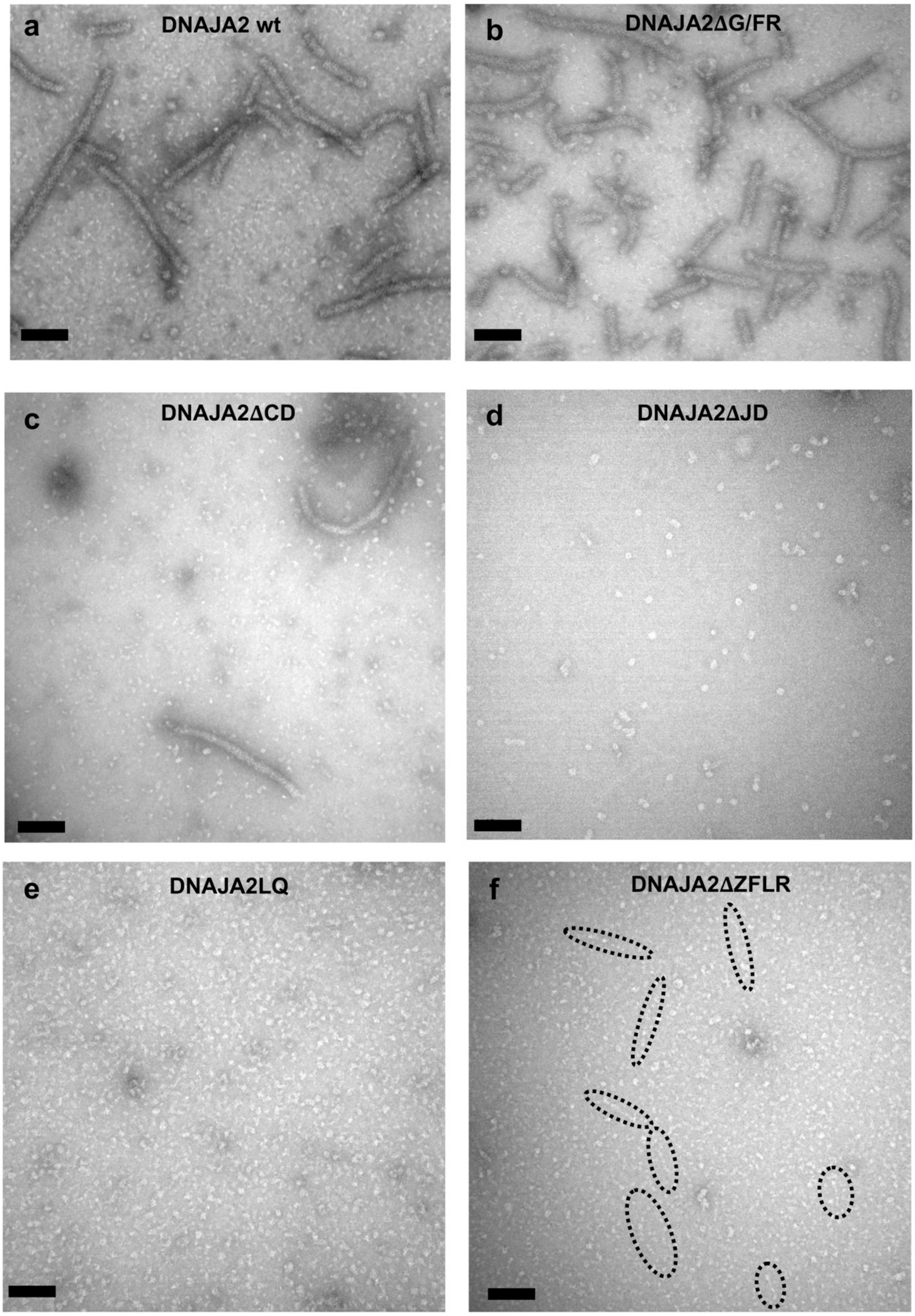

Supplementary Figure 3. Negative staining microscopy images of the samples analysed in this work. (a) DNAJA2wt. **(b)** DNAJA2ΔG/FR. **(c)** DNAJA2ΔCD. **(d)** DNAJA2ΔJD. **(e)** DNAJA2LQ and **(f)** DNAJA2ΔZFLR. Filaments are encircled. Representative EM images observed in three independent protein preparations. Scale bars represent 100 nm.

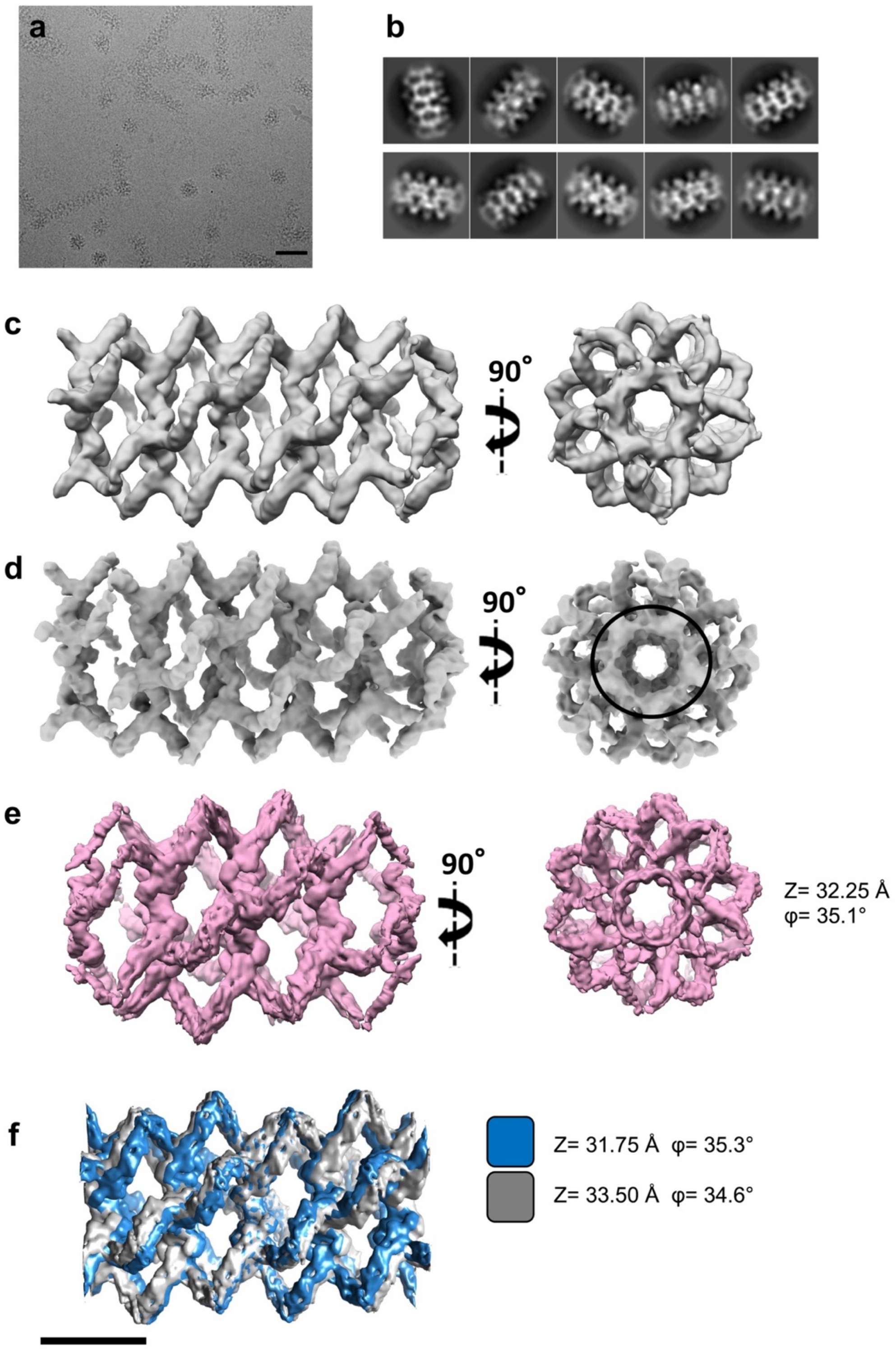

Supplementary Figure 4. Architecture of DNAJA2 oligomers. (a) Representative field of a CryoEM image of DNAJA2wt. Similar CryoEM images were obtained in three independent protein preparations. Bar indicates 500 Å. **(b)** Selected classes of a maximum-likelihood 2D classification of the particles. **(c)** Side and top views of the 3D reconstruction of DNAJA2wt without symmetry imposition. **(d)** Side and top views of the D5-symmetry imposed DNAJA2 map. **(e)** Two different views of the 3D reconstruction of DNAJA2ΔG/FR applying helical symmetry showing Z and φ parameters. **(f)** Two different degrees of extension and rotation observed in the helical map. Bar indicates 200 Å.

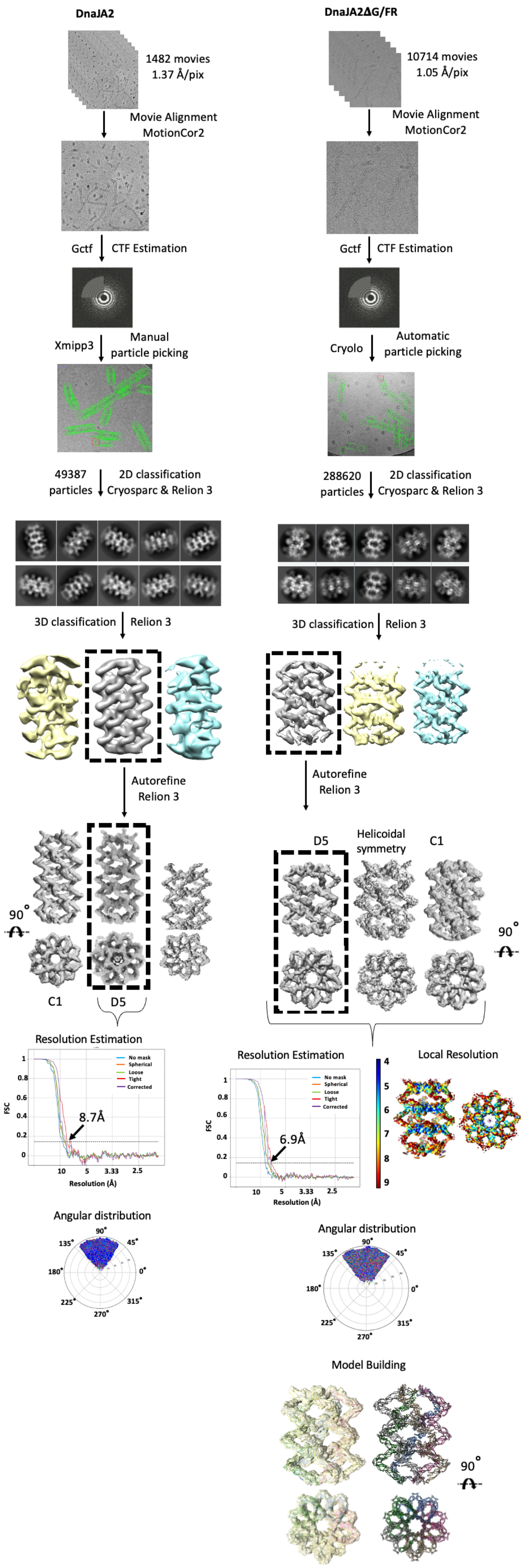

DnaJA2
DnaJA2ΔG/FR
1482 movies
1.37 Å/pix
10714 movies
1.05 Å/pix
Movie Alignment
MotionCor2
Gctf
CTF Estimation
Xmipp3
Manual
particle picking
Cryolo
Automatic
particle picking
49387
particles
288620
particles
2D classification
Cryosparc & Relion 3
3D classification
Relion 3
Autorefine
Relion 3
90°
C1
D5
Helicoidal
symmetry
Resolution Estimation
Local Resolution
No mask
Spherical
Loose
Tight
Corrected
FSC
Resolution (Å)
8.7Å
6.9Å
Angular distribution
Model Building

Supplementary Figure 5. Workflow of the 3D reconstruction process of DNAJA2wt and DNAJA2ΔG/FR. Main steps of the image processing, highlighting the 3D classifications and the refinements performed using different symmetries. The Fourier Shell Correlation (FSC) curves resolutions of the final maps estimated using the Gold-standard FSC criterion are also shown, together with the angular distribution of the particles used in the final steps of the refinements.

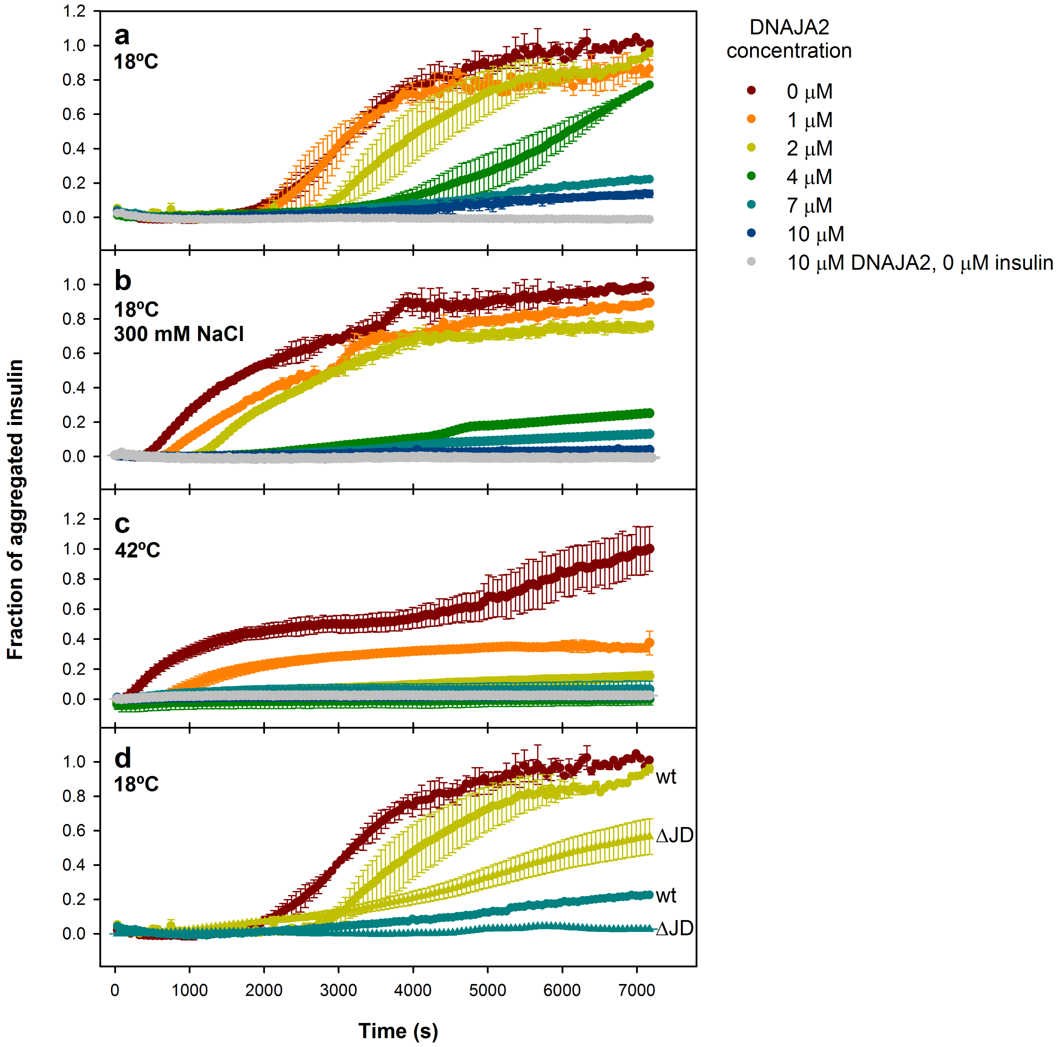

Supplementary Figure 6. Holding activity of DNAJA2wt under conditions that favour the oligomer or the dimer. Insulin (final concentration 45 μM) was incubated 30 min with increasing concentrations of DNAJA2wt at (**a**) 18 °C/0 mM NaCl, (**b**) 18 °C/300 mM NaCl or (**c**) 42 °C/0 mM NaCl. Aggregation of insulin was initiated by the addition of DTT (final concentration 15 mM). The kinetics of insulin aggregation was recorded by light scattering at 400 nm in 40 mM Hepes/KOH pH 7.5. As a control, the scattering of 10 μM DNAJA2 in the absence of insulin was monitored under each of the experimental conditions used (grey lines). (**d**) Comparison of the holding activity of DNAJA2wt and DNAJA2ΔJD at 18 °C/0 mM NaCl. Colour code indicated in the Figure. Data are means ± SD of three independent experiments.

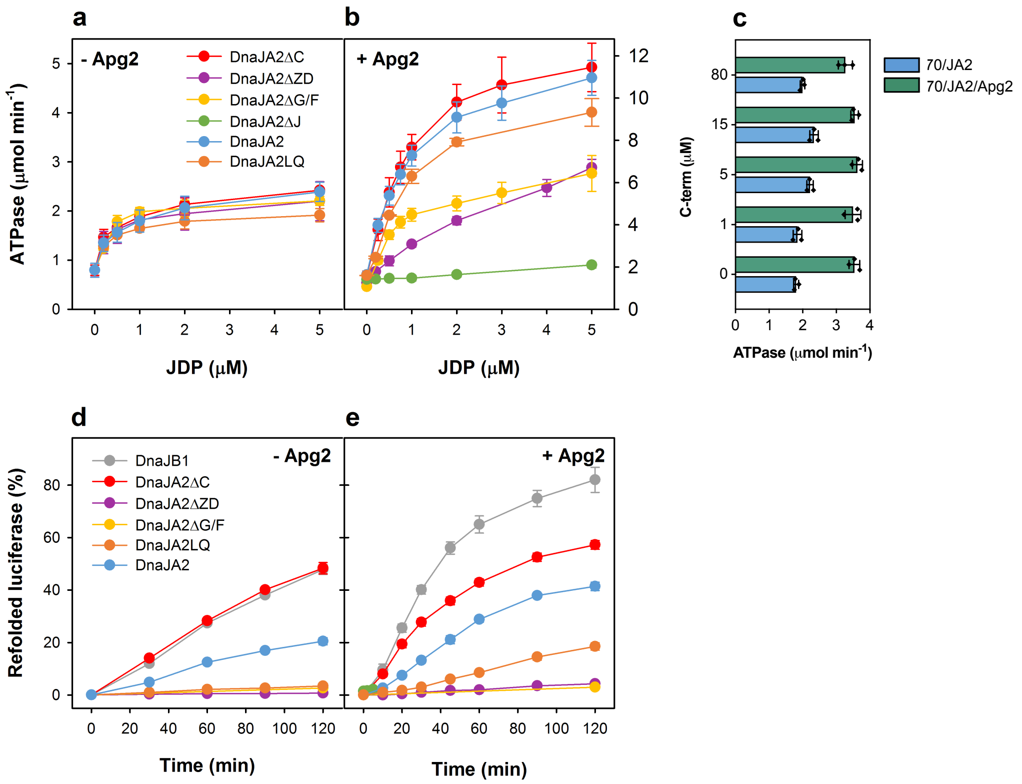

Supplementary Figure 7. Functional characterization of DNAJA2 and its mutants. Stimulation of the ATPase activity of Hsc70 (2 µM) by increasing concentrations of DNAJA2wt or its mutants in the absence (**a**) and presence (**b**) of 0.4 µM Apg2. (**c**) Purified CD did not activate Hsc70 regardless of the presence of Apg2. Reactivation of chemically denatured luciferase (20 nM) aggregates by 2 µM Hsc70, 0.5 µM DnaJB1, DNAJA2 or its variants in the absence (**d**) or presence (**e**) of 0.4 µM Apg2. The colour code used is indicated. Data are mean ± SD of three independent experiments.

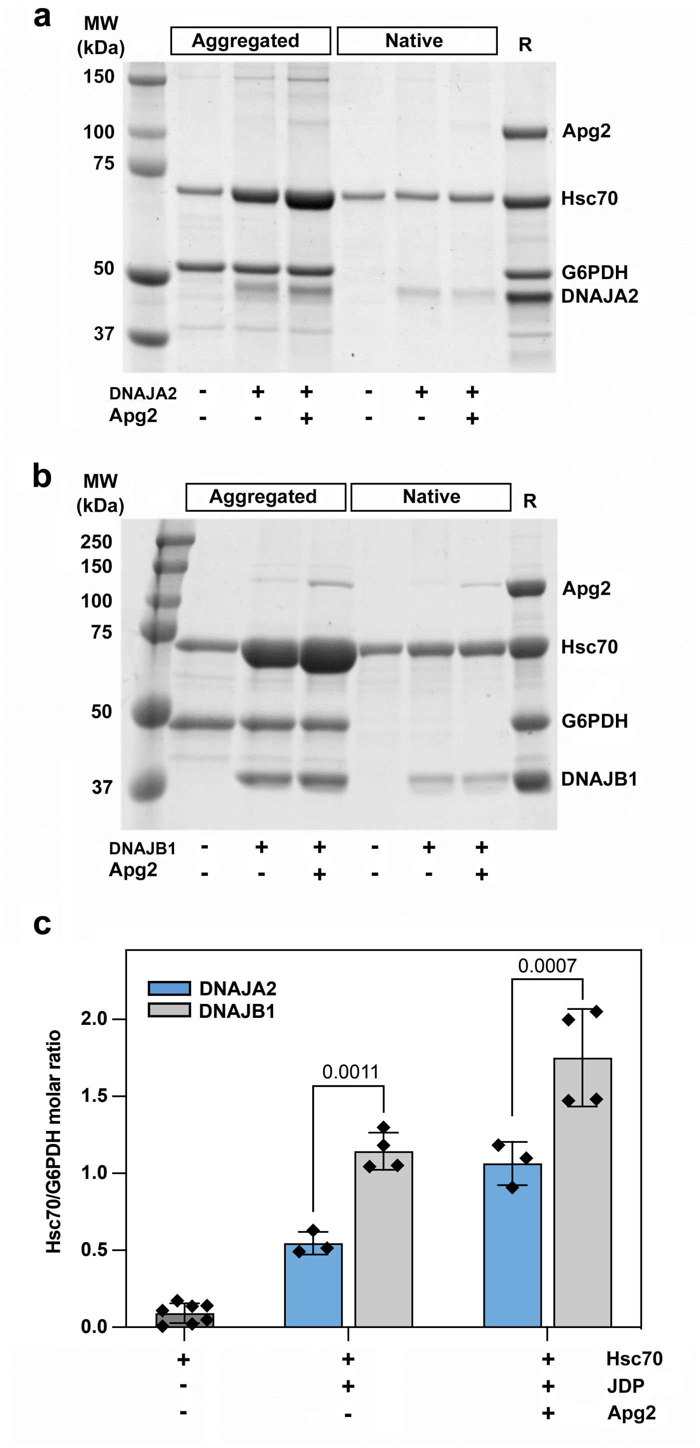

Supplementary Figure 8. DnaJB1 recruits Hsc70 to G6PDH aggregates more efficiently than DNAJA2. SDS-PAGE gels of (**a**) DNAJA2- or (**b**) DnaJB1-mediated (1 µM) recruitment of Hsc70 (2 µM) to G6PDH (0.4 µM) aggregates in the absence or presence of Apg2 (0.4 µM). Reference lanes (R) contain 1 µg of each chaperone and native G6PDH. Left, molecular weight markers (MW). (**c**) Estimation of the Hsc70/G6PDH molar ratios using data shown in panels A-B for the different chaperone mixtures. Data correspond to mean ± SD of three (JA2) or four (JB1) independent experiments. Significance was evaluated using a two-tailed, one-way analysis of variance (ANOVA) and Tukey´s multiple comparison, and *p* values are shown in c.

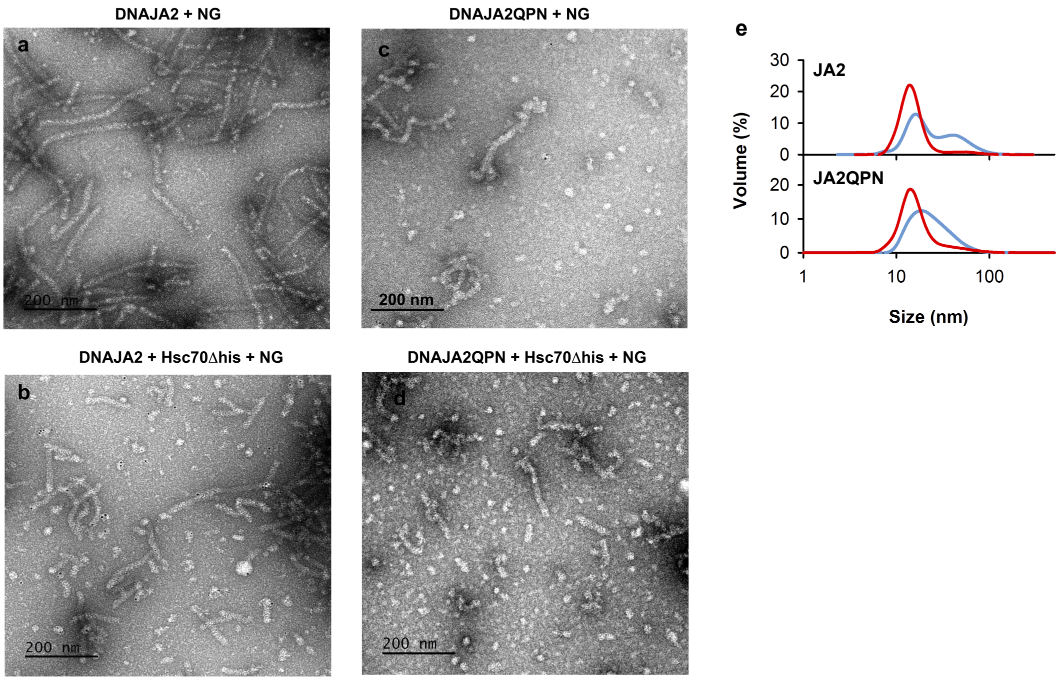

Supplementary Figure 9. Binding of Hsc70 to DNAJA2 or DNAJA2QPN assemblies followed by chaperone labelling. Negative staining EM images of samples containing 15 µM DNAJA2wt (**a, b**) or DNAJA2QPN (**c, d**) incubated 90 s in refolding buffer at 4 °C without (**a, c**) or with (**b, d**) 7 µM His-tagged Hsc70Δlid. Afterwards, Ni-NTA-nanogold beads were added to the samples and incubated 30 min at RT. After additional washing steps with imidazole and water, the samples were imaged. Similar images were obtained in three different protein preparations. (**e**) DLS of 30 µM DNAJA2wt (top panel) or DNAJA2QPN (bottom panel) recorded in refolding buffer at 25 °C (blue) or 40 °C (red).

Supplementary Table 1. Data collection parameters for DNAJA2wt and DNAJA2ΔG/FR

Data collection	**DNAJA2 wt**<br>EMD-14729 (C1)<br>EMD-14727 (D5)	**DNAJA2ΔG/FR**<br>EMD-14706 (C1)<br>EMD-14736 (D5)
**Microscope**	Talos Arctica	Titan Krios
**Voltage (keV)**	200	300
**Detector**	Falcon III	K3
**Nominal magnification**	73,000x	130,000x
**Pixel size (Å)**	1.37	1.053
**Defocus range (µm)**	-1.2 to -3.4	-1.2 to -2.6
**Exposure time (s)**	1	2
**Electron dose (e^-/Å^2)**	61	30
**Frames**	60	40
**Dose/frame (e^-/Å^2)**	1	0.75
**Movies (no.)**	1482	10714
**Initial particles (no.)**	49387	288620
**Final particles (no.)**	11388	47396
**Final resolution (Å)**	C1 / D5	C1 / D5
	12.7 / 8.7	9.2 / 6.9

Supplementary Table 2. DnaJA2ΔG/FR model refinement and validation statistics related to Figure 3.

**Map**<br>**Model**	**DnaJA2ΔG/FR**<br>EMDB-14736<br>PDB-7ZHS	
**Composition (#)**		
Chains	40	
Atoms	67465 (Hydrogens: 0)	
Residues	Protein: 8695 Nucleotide: 0	
Water	0	
Ligands	ZN: 80	
**Bonds (RMSD)**		
Length (Å) (# > 4σ)	0.010 (76)	
Angles (°) (# > 4σ)	1.606 (426)	
**MolProbity score**	2.75	
**Clash score**	49.53	
**Ramachandran plot (%)**		
Outliers	0.93	
Allowed	3.59	
Favored	95.48	
**Rotamer outliers (%)**	2.23	
**Cβ outliers (%)**	0.00	
**Peptide plane (%)**		
Cis proline/general	0.0/0.0	
Twisted proline/general	0.0/0.0	
**CaBLAM outliers (%)**	2.23	
**ADP (B-factors)**		
Iso/Aniso (#)	67465/0	
min/max/mean		
Protein	39.53/440.00/354.69	
Nucleotide	---	
Ligand	30.78/343.24/144.89	
Water	---	
**Occupancy**		
Mean	1.00	
occ = 1 (%)	100.00	
0 < occ < 1 (%)	0.00	
occ > 1 (%)	0.00	
**Data**		
**Box**		
Lengths (Å)	238.20, 242.42, 271.93	
Angles (°)	90.00, 90.00, 90.00	
**Supplied Resolution (Å)**	8.0	
**Resolution Estimates (Å)**	Masked	Unmasked
d FSC (half maps; 0.143)	7.9	8.2
d 99 (full/half1/half2)	9.1/6.5/6.4	8.4/4.8/4.8
d model	9.5	9.5

d FSC model (0/0.143/0.5)	6.9/7.8/8.7	6.9/7.9/8.9
**Map min/max/mean**	-0.08/0.44/0.02	
**Model vs. Data**		
**CC (mask)**	0.81	
**CC (box)**	0.86	
**CC (peaks)**	0.72	
**CC (volume)**	0.77	
**Mean CC for ligands**	0.76	

Supplementary Table 3: Crosslinks detected upon treating DNAJA2wt and DNAJA2ΔG/FR with BS3, under conditions that favour the oligomer population (low salt concentration, 25 °C). The crosslinked residues in bold and the distances between their Cα estimated in the proposed structural model are also shown. Distances between crosslinked residues were estimated considering that they could be in the same monomer (intra-monomer), in different monomers of a dimer (intra-dimer), or in neighboring dimers (inter-dimer). The inter-dimer one corresponds to the shortest distance between crosslinked residues of a given dimer and the four dimers surrounding it. Crosslinks were considered inter-dimer when the distance between residues of adjacent dimers was below or around 30 Å and the intra-dimer or intra-monomer distances were significantly larger. The most likely contacts according to the structural model are shown in a green box. Those marked with an asterisk are shown in Figs. 3f and 4d. The score and FDR values correspond to DNAJA2wt/DNAJA2ΔG/FR when the crosslinks are detected in both protein species. Distances cannot be estimated when one of the crosslinked residues belongs to the CD, and therefore assignment of these crosslinks is not feasible.

Domains	Crosslinked peptide	Pos 1	Pos 2	WT	ΔG/F	Cα-Cα (Å)			Score	FDR (%)
						Inter-dimer	Intra-dimer	Intra-monomer		
JD-JD	^{1}**M**ANVADTK^{8}-^{1}**M**ANVADTK^{8}	1	1	yes	yes	32	106	--	237/154	0/0
	^{1}**M**ANVADTK^{8}-^{26}**K**AYR^{29}	1	26	yes	no	30	103	20	172	0
	^{1}**M**ANVADTK^{8}-^{47}F**K**EISFAYEVLSNPEK^{62}	1	48	no	yes	40	88	22	144	0
	^{1}**M**ANVADTK^{8}-^{49}EISFAYEVLSNPE**K**R^{63}	1	62	no	yes	35	102	15	133	0
	^{1}**M**ANVADTK^{8}-^{64}EL**Y**DRYGEQGLR^{75}	1	66	no	yes	36	98	13	69	0
	^{47}F**K**EISFAYEVLSNPEKR^{63}-^{69}**Y**GEQGLR^{75}	48	69	no	yes	36	80	16	136	0
JD-CTDI	^{1}**M**ANVADTK^{8}-^{113}GEDMMHPL**K**VSLEDLYNGK^{131} **(*)**	1	121	no	yes	26	86	53	121	0
	^{1}**M**ANVADTK^{8}-^{132}TT**K**LQLSK^{139}	1	134	yes	yes	33	97	49	188/132	0/0
	^{1}**M**ANVADTK^{8}-^{210}VI**K**EVK^{215} (*)	1	212	yes	no	20	91	44	179	0
	^{1}**M**ANVADTK^{8}-^{213}EV**K**ILEVHVDK^{223} (*)	1	215	no	yes	24	96	44	149	0
	^{31}LA**K**EYHPDK^{39}-^{224}GM**K**HGQR^{230} **(*)**	33	226	no	yes	18	68	36	104	0

	^{34}EYHPD**K**NPNAGDKFK^{48}-^{112}RGEDMMHPL**K**VSLEDLYNGK^{131}	39	121	no	yes	28	58	43	129	0
	^{34}EYHPD**K**NPNAGDKFK^{48}-^{213}EV**K**ILEVHVDK^{223}	39	215	no	yes	31	67	46	54	0
	^{34}EYHPD**K**NPNAGDKFK^{48}-^{224}GM**K**HGQR^{230} (*)	39	226	no	yes	23	62	35	104	0
JD-ZFD	^{1}**M**ANVADTK^{8}-^{140}NVLBSABSGQGG**K**SGAVQK^{158} (*)	1	152	no	yes	14	100	37	46	0
	^{1}**M**ANVADTK^{8}-^{153}**S**GAVQK^{158} (*)	1	153	yes	no	17	99	35	200	0
	^{1}**M**ANVADTK^{8}-^{153}SGAVQ**K**^{158}	1	158	yes	no	26	88	44	154	1.1
	^{1}**M**ANVADTK^{8}-^{153}SGAVQ**K**BSABR^{163}	1	158	no	yes	26	88	44	74	0
	^{1}**M**ANVADTK^{8}-^{173}QLAPGMVQQMQSVBSDBNGEGEVINE**K**DR^{201} (*)	1	199	no	yes	11	100	46	130	0
	^{1}**M**ANVADTK^{8}-^{204}**K**BEGK^{208}	1	204	no	yes	13	96	46	133	0
	^{34}EYHPDKNPNAGD**K**FK^{48}-^{140}NVLBSABSGQGG**K**SGAVQK^{158} (*)	46	152	no	yes	20	81	40	60	0
	^{47}F**K**EISFAYEVLSNPEKR^{63}-^{140}NVLBSABSGQGG**K**SGAVQK^{158} (*)	48	152	no	yes	20	84	36	98	0
CTDI-ZFD	^{112}RGEDMMHPL**K**VSLEDLYNGK^{131}-^{140}NVLB**S**ABSGQGGK^{152} (*)	121	144	no	yes	23	85	34	30	0
	^{113}GEDMMHPL**K**VSLEDLYNGK^{131}-^{204}**K**BEGK^{208} (*)	121	204	no	yes	22	84	34	108	0
	^{213}EV**K**ILEVHVDK^{223}-^{140}NVLB**S**ABSGQGGK^{152}	215	144	no	yes	31	99	15	118	0
	^{213}EV**K**ILEVHVDK^{223}-^{204}**K**BEGKK^{209}	215	204	no	yes	28	97	17	94	0
CTDI-CTDI	^{122}VSLEDLYNGK**T**TK^{134}-^{122}VSLEDLYNGK**T**TK^{134} (*)	132	132	no	yes	18	79	--	57	0
	^{132}TT**K**LQLSK^{139}-^{132}TT**K**LQLSK^{139} (*)	134	134	yes	yes	11	82	--	151/141	1.1/0
	^{209}KVI**K**EVK^{215}-^{210}VI**K**EVK^{215} (*)	212	212	yes	yes	18	90	--	184/127	0
CTDI-CTDII	^{216}ILEVHVD**K**GMK^{226}-^{322}NPFE**K**GDLYIK^{332}	223	326	no	yes	43	63	10	88	0
ZFLR-ZFLR	^{140}NVLBSABSGQGG**K**SGAVQK^{158}-^{153}SGAVQ**K**BSABR^{163}	152	158	no	yes	37	101	14	84	0
	^{153}SGAVQ**K**BSABR^{163}-^{153}SGAVQ**K**BSABR^{163} (*)	158	158	no	yes	30	95	--	91	0

CTDII-CTDII	^{273}IGLVEALBGFQF**T**FKHLDGR^{292}-^{298}YPPG**K**VIEPGBVR^{310}	285	302	no	yes	61	35	19	74	0
	^{298}YPPG**K**VIEPGBVR^{310}-^{322}NPFE**K**GDLYIK^{332}	302	326	no	yes	64	47	26	110	0
JD-CD	^{1}**M**ANVADTK^{8}-^{381}G**S**GGGQR^{387}	1	382	yes	no				107	0
	^{1}**M**ANVADTK^{8}-^{389}EA**Y**NDSSDEESSSHHGPGVQBAHQ^{412}	1	391	no	yes				120	0
	^{1}**M**ANVADTK^{8}-^{389}EAYNDSSDEE**S**SSHHGPGVQBAHQ^{412}	1	399	no	yes				120	0
	^{1}**M**ANVADTK^{8}-^{388}REAYNDSSDEESS**S**HHGPGVQBAHQ^{412}	1	401	no	yes				63	0
	^{31}LA**K**EYHPDKNPNAGDK^{46}-^{381}G**S**GGGQR^{387}	33	382	no	yes				77	0
	^{34}EYHPD**K**NPNAGDK^{46}-^{381}G**S**GGGQR^{387}	39	382	no	yes				94	0
	^{49}EISFAYEVLSNPE**K**R^{63}-^{389}EA**Y**NDSSDEESSSHHGPGVQBAHQ^{412}	62	391	no	yes				73	0
CTDI-CD	^{112}RGEDMMHPL**K**VSLEDLYNGK^{131}-^{381}G**S**GGGQR^{387}	121	382	no	yes				69	0
	^{132}TT**K**LQLSK^{139}-^{389}EA**Y**NDSSDEESSSHHGPGVQBAHQ^{412}	134	391	no	yes				120	0
	^{153}SGAVQ**K**^{158}-^{381}G**S**GGGQR^{387}	158	391	yes	no				125	5
	^{216}ILEVHVD**K**GMKHGQR^{230}-^{381}G**S**GGGQR^{387}	223	382	no	yes				109	0
CTDII-CD	^{293}QIVV**K**YPPGK^{302}-^{389}EA**Y**NDSSDEESSSHHGPGVQBAHQ^{412}	297	391	no	yes				90	0
	^{298}YPPG**K**VIEPGBVR^{310}-^{381}G**S**GGGQR^{387}	303	382	no	yes				134	0
	^{298}YPPG**K**VIEPGBVR^{310}-^{389}EA**Y**NDSSDEESSSHHGPGVQBAHQ^{412}	303	391	no	yes					0
	^{298}YPPG**K**VIEPGBVR^{310}-^{389}EAYNDSSDEE**S**SSHHGPGVQBAHQ^{412}	303	399	no	yes				66	0
	^{322}NPFE**K**GDLYIK^{332}-^{389}EA**Y**NDSSDEESSSHHGPGVQBAHQ^{412}	326	391	no	yes				57	0

Supplementary references

1. Jumper. J., Evans. R., Pritzel. A., Green. T., Figurnov, M., Ronneberger, O., Tunyasuvunakool, K., Bates, R., Žídek, A., Potapenko, A., Bridgland, A., Meyer, C., Kohl, SAA., Ballard, A.J., Cowie, A., Romera-Paredes, B., Nikolov, S., Jain, R., Adler. J., Back, T., Petersen, S., Reiman, D., Clancy, E., Zielinski, M., Steinegger, M., Pacholska, M., Berghammer, T., Bodenstein, S., Silver, D., Vinyals, O., Senior, A.W., Kavukcuoglu, K., Kohli, P., & Hassabis, D. (2021) *Nature 596*, 583-589. doi: 10.1038/s41586-021-03819-2

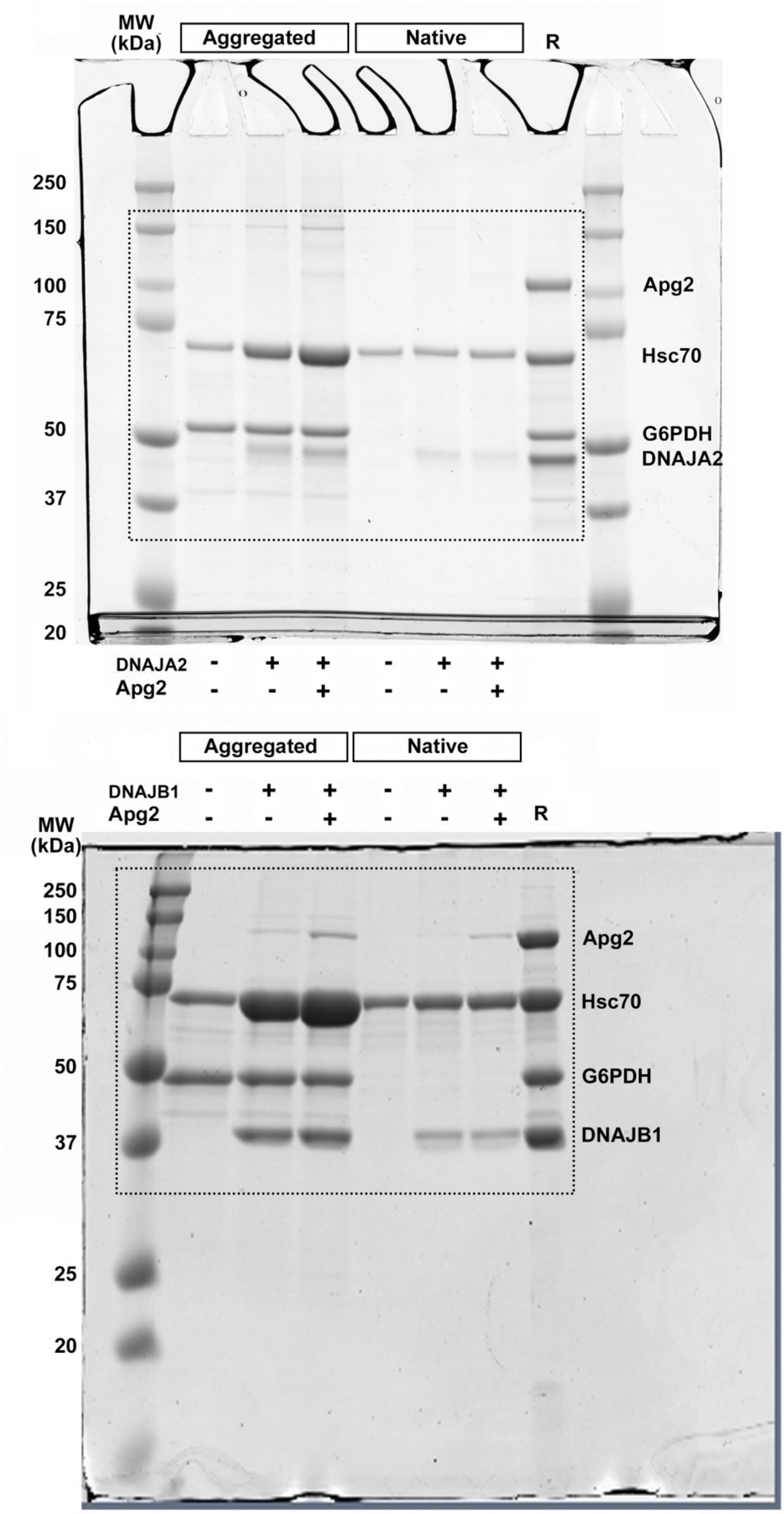

Uncropped gels corresponding to Supplementary Figure 8.
